# Supplementary material for: Machine learning assisted composition design of high-entropy Pb-free relaxors with giant energy-storage
Source: Nat Commun. 2025 Feb 1;16:1254. doi: 10.1038/s41467-025-56443-3 (PMC11787375; doi:10.1038/s41467-025-56443-3)
Supplement: Supplementary file 1 — Supplementary Information [file 41467_2025_56443_MOESM1_ESM.pdf]

Machine learning assisted composition design of high-entropy Pb-free relaxors with giant energy-storage

Xingcheng Wang[1,#], Ji Zhang[2,#], Xingshuai Ma[1,#], Huajie Luo[1], Laijun Liu[3], Hui Liu[1,*], and Jun Chen[1,*]

[1]Beijing Advanced Innovation Center for Materials Genome Engineering, Department of Physical Chemistry, University of Science and Technology Beijing, Beijing 100083, China

[2]School of Materials Science and Engineering, Nanjing University of Science and Technology, Nanjing, Jiangsu 210094, China

[3]College of Materials Science and Engineering, Guilin University of Technology, Guilin 541004, China

[#]Xingcheng Wang, Ji Zhang, and Xingshuai Ma contribute equally to this work.

*Corresponding author. Email: huiliu@ustb.edu.cn and junchen@ustb.edu.cn

Methods

Machine learning

1. Dataset and feature pool: The dataset of BNT-based ceramics investigated in this study was collected from the published literature (see Supplementary Table 1 for detailed data). We selected various descriptors in terms of structural and geometrical properties, electron and charge distribution properties, electronegativity, and nucleus properties (see Supplementary Table 2 for details) and generated feature values matrix for samples in the dataset by weighted summation.

2. Feature selection: Pearson correlation analysis was used to identify key features of the machine learning model. The Pearson correlation coefficient (r) is mathematically expressed as:

$$r = \frac{\sum_{i=1}^{n}(x_i - \bar{x})(y_i - \bar{y})}{\sqrt{\sum_{i=1}^{n}(x_i - \bar{x})^2 \sum_{i=1}^{n}(y_i - \bar{y})^2}} \tag{1}$$

Here, n denotes the number of features, x_i and y_i represent two distinct features, with $\bar{x}$ and $\bar{y}$ being their respective means. The higher the value of r, the more strongly the two features are linearly related. Setting 0.95 is a threshold for the correlation coefficient to determine if features are too similar to each other. If the correlation coefficient of two features is greater than or equal to this threshold, they are considered highly correlated. For the group of features with high correlation, one of the features is retained and the other related features are removed. The goal of this step is to reduce the dimensionality of the feature space while reducing the risk of overfitting the model. After screening by Pearson's correlation coefficient, the remaining features are usually those that have a strong correlation with the target variable (y) and a low correlation between them and other features. These features retain more useful information and help to improve the performance of the model. Subsequently, through the combination of recursive feature elimination and cross-

validation, the best subset of features is identified by eliminating one feature at a time and iterating continuously, using 10-fold cross-validation to evaluate the model performance after each feature elimination.

3. Model selection: The performance of various machine learning regression algorithms, including Random Forest (RF), Gradient Boosted Regressor (GBR), Decision Tree Regressor (DTR), Radial Basis Function Kernel Support Vector Regression (SVR.rbf), and Bayesian Ridge Regression (BR), was evaluated using *k*-fold cross-validation (CV). The definition is as follows:

$$CV = \sqrt{\frac{1}{n}\sum_{i=1}^{n}(y_i - \hat{y_i})^2} \quad (2)$$

where *n* is the number of observations in the training dataset, y_i is the true value, and $\hat{y_i}$ is the predicted value. In searching for suitable model hyperparameters, we traverse the given hyperparameter space through a grid search, train each set of possible hyperparameter combinations and evaluate their performance on the validation set to select the set of hyperparameters and find the best model.

4. Regression fitting: Firstly, the feature matrix and target variables are extracted from the given dataset. Next, a random forest regression model was used for training and predictions were made on the test set. To assess the model performance, we calculated the coefficient of determination (R^2) and the mean absolute error (MAE) to evaluate the model fit.

5. Prediction: The new composition space is constructed based on the given elemental ratios and valence information, which needs to satisfy the sum of the valences of the *A*-site and *B*-site elements to be +6, and ultimately to meet the requirements for the formation of the perovskite structure ABO_3. After screening out the material combinations that meet the requirements, they are transformed into the corresponding feature values matrix. Subsequently, a trained Random Forest model is used to predict the new data, and all the cross-validated predictions (for each fold) are

averaged to obtain the final predicted values to reduce the bias of the model, thus providing guidance for the material design.

6. Dataset sample size reasonableness validation: In order to verify the effectiveness of a relatively small amount of data for machine learning, the learning curve of the optimized random forest model showing the training scores for different sample sizes in the training set is provided in Supplementary Figure 1. It can be seen that when the sample size in the training set exceeds 80, the validation set score reaches a high and stable level, and the model is not affected by overfitting or underfitting. This is a good indication that the model can learn effective information and make accurate predictions even with a relatively small amount of data.

7. Data and code available: The feature values for the dataset can be found in the supplementary data. The related codes have been uploaded to Code Ocean.

Sample fabrication

Ceramics samples of predicted high-entropy compositions of $(Bi_{0.36}Na_{0.34}La_{0.13}Sr_{0.17})(Ti_{0.86}Ta_{0.01}Mg_{0.08}Hf_{0.05})O_3$ (Sample A), $(Bi_{0.36}Na_{0.33}La_{0.03}Ca_{0.28})(Ti_{0.82}Zr_{0.1}Mg_{0.03}Hf_{0.05})O_3$ (Sample B), $(Bi_{0.37}Na_{0.34}La_{0.14}Ca_{0.15})(Ti_{0.86}Ta_{0.01}Mg_{0.09}Hf_{0.04})O_3$ (Sample C) and $(Bi_{0.36}Na_{0.33}La_{0.13}Sr_{0.18})(Ti_{0.86}Zr_{0.01}Mg_{0.08}Hf_{0.05})O_3$ (Sample D) were fabricated by a solid-state sintering method. High-purity Bi_2O_3 (99.99%), Na_2CO_3 (99.99%), La_2O_3 (99.99%), $SrCO_3$ (99.95%), $CaCO_3$ (99%), TiO_2 (99.9%), ZrO_2 (99.99%), Ta_2O_5 (99.5%), MgO (99.99%), and HfO_2 (99.9%) were used as the raw materials. These weighed powders were mixed with anhydrous ethanol and then planetary-milled for 24 hours. The mixed powders were calcined at 820 °C for 2 h and sintered at 1100 - 1120 °C for 2 h.

Structural Characterizations

X-ray diffraction (XRD) patterns were measured by using an X-ray diffractometer (Rigaku, Japan) with a Co radiation. The grain morphology and elemental distribution of the ceramic samples were observed by scanning electron microscopy (SEM,

LEO1530, Germany). Atomic-scale high-angle dark-field annular (HAADF) STEM images were recorded using a scanning transmission microscopy (JEM-F200, JEOL, Japan) equipped with probe and image aberration-correctors operated at 200 kV. To remove noise, all HAADF-STEM images were Fourier-filtered using a lattice mask, and the atomic positions were determined by 2D Gaussian fitting as well as a customized MATLAB script to extract the atomic displacement vector.

Electrical Properties Measurements

The bulk ceramic samples were polished to a thickness of approximately 50 μm and an Au electrode of about 0.8 mm^2 (diameter about 1mm) was used to measure energy storage and charging/discharging properties. A ferroelectric analyzer (aix ACCT, TF analyzer 1000, Germany) was employed to measure the bipolar *P-E* loop at low electric fields and the unipolar *P-E* loop to obtain the energy storage density and efficiency. Dielectric-temperature spectra were measured using a dielectric spectroscopy measurement system (LDM-500, China), while electrochemical impedance was measured using a precision impedance analyzer (Agilent 4294A, USA). The overdamped charge/discharge tests were measured using the Tongguo (TG) technique (CFD-003, China).

Absorption Spectrum Measurements

The UV-Vis diffuse reflectance spectra (DRS) of the test samples were tested using a hitachi UH4150 (Japan), baseline scanned using a barium sulphate standard white plate, and the spectra of the samples were collected after correction.

Mechanical Performance Measurements

The Vickers hardness values of the samples were measured using a Wolpert Wilson instrument (401MVD, China). Each indentation was performed with an applied load of 9.8 N and a dwell time of 15 seconds. A total of three indentations were made on each sample, and the average value was calculated.

Supplementary Table 1. The original data of machine learning. 121 sets of original data.

Composition	W_{rec} (J cm^{-3})	E_B (kV mm^{-1})
$0.76Bi_{0.5}Na_{0.5}TiO_3$–$0.24SrTiO_3$	0.67	10
$0.98(0.76Bi_{0.5}Na_{0.5}TiO_3$–$0.24SrTiO_3)$-$0.02AgNbO_3$	1.26	10
$0.97(0.76Bi_{0.5}Na_{0.5}TiO_3$–$0.24SrTiO_3)$-$0.03AgNbO_3$	1.28	10
$0.95(0.76Bi_{0.5}Na_{0.5}TiO_3$–$0.24SrTiO_3)$-$0.05AgNbO_3$	1.42	10
$0.93(0.76Bi_{0.5}Na_{0.5}TiO_3$–$0.24SrTiO_3)$-$0.07AgNbO_3$	1.29	10
$0.91(0.76Bi_{0.5}Na_{0.5}TiO_3$–$0.24SrTiO_3)$ -$0.09AgNbO_3$	1.19	10
$0.8Bi_{0.5}Na_{0.5}TiO_3$-$0.2Sr_{0.7}La_{0.2}TiO_3$	1.5	15
$0.7Bi_{0.5}Na_{0.5}TiO_3$-$0.3Sr_{0.7}La_{0.2}TiO_3$	2.44	18
$0.65Bi_{0.5}Na_{0.5}TiO_3$-$0.35Sr_{0.7}La_{0.2}TiO_3$	2.96	23
$0.55Bi_{0.5}Na_{0.5}TiO_3$-$0.45Sr_{0.7}La_{0.2}TiO_3$	4.14	32
$0.5Bi_{0.5}Na_{0.5}TiO_3$-$0.5Sr_{0.7}La_{0.2}TiO_3$	3.71	34
0.76 $Bi_{0.5}Na_{0.5}TiO_3$-$0.24NaNbO_3$	1.45	15
$0.92(0.76Bi_{0.5}Na_{0.5}TiO_3$-$0.24NaNbO_3)$-$0.08Sr_{0.7}Bi_{0.2}TiO_3$	1.55	15
$0.9(0.76Bi_{0.5}Na_{0.5}TiO_3$-$0.24NaNbO_3)$-$0.1Sr_{0.7}Bi_{0.2}TiO_3$	1.97	15
$0.88(0.76Bi_{0.5}Na_{0.5}TiO_3$-$0.24NaNbO_3)$-$0.12Sr_{0.7}Bi_{0.2}TiO_3$	1.24	15
$0.9[0.8Bi_{0.5}Na_{0.5}TiO_3$-$0.2Ba(Zr_{0.3}Ti_{0.7})O_3]$-$0.1Sr_{0.7}La_{0.2}TiO_3$	1.25	10
$0.8[0.8Bi_{0.5}Na_{0.5}TiO_3$-$0.2Ba(Zr_{0.3}Ti_{0.7})O_3]$-$0.2Sr_{0.7}La_{0.2}TiO_3$	1.04	10
$0.7[0.8Bi_{0.5}Na_{0.5}TiO_3$-$0.2Ba(Zr_{0.3}Ti_{0.7})O_3]$-$0.3Sr_{0.7}La_{0.2}TiO_3$	0.94	10
$0.6[0.8Bi_{0.5}Na_{0.5}TiO_3$-$0.2Ba(Zr_{0.3}Ti_{0.7})O_3]$-$0.4Sr_{0.7}La_{0.2}TiO_3$	0.68	10
$(Bi_{0.5}Na_{0.5})_{0.65}Sr_{0.35}TiO_3$	2.52	28
$0.95(Bi_{0.5}Na_{0.5})_{0.65}Sr_{0.35}TiO_3$-$0.05Bi(Mg_{0.5}Zr_{0.5})O_3$	5.25	44
$0.9(Bi_{0.5}Na_{0.5})_{0.65}Sr_{0.35}TiO_3$-$0.1Bi(Mg_{0.5}Zr_{0.5})O_3$	8.46	52
$0.85(Bi_{0.5}Na_{0.5})_{0.65}Sr_{0.35}TiO_3$-$0.15Bi(Mg_{0.5}Zr_{0.5})O_3$	5.15	34
$0.85Bi_{0.5}Na_{0.5}TiO_3$-$0.15NaNbO_3$	1.12	16
$0.72Bi_{0.5}Na_{0.5}TiO_3$-$0.28SrTiO_3$	0.62	12
$0.99(0.72Bi_{0.5}Na_{0.5}TiO_3$-$0.28SrTiO_3)$-$0.01Ba_{1/3}Bi_{2/3}Nb_{2/3}O_3$	1.52	12
$0.98(0.72Bi_{0.5}Na_{0.5}TiO_3$-$0.28SrTiO_3)$-$0.02Ba_{1/3}Bi_{2/3}Nb_{2/3}O_3$	1.6	12
$0.97(0.72Bi_{0.5}Na_{0.5}TiO_3$-$0.28SrTiO_3)$-$0.03Ba_{1/3}Bi_{2/3}Nb_{2/3}O_3$	1.33	12
$0.96(0.72Bi_{0.5}Na_{0.5}TiO_3$-$0.28SrTiO_3)$-$0.04Ba_{1/3}Bi_{2/3}Nb_{2/3}O_3$	1.17	12
$0.95(0.72Bi_{0.5}Na_{0.5}TiO_3$-$0.28SrTiO_3)$-$0.05Ba_{1/3}Bi_{2/3}Nb_{2/3}O_3$	0.98	12
$0.75Na_{0.5}Bi_{0.5}TiO_3$-$0.25SrTiO_3$	2.24	20
$0.9(0.75Na_{0.5}Bi_{0.5}TiO_3$-$0.25SrTiO_3)$-$0.1CaTi_{0.875}Nb_{0.1}O_3$	3.36	24
$0.8(0.75Na_{0.5}Bi_{0.5}TiO_3$-$0.25SrTiO_3)$-$0.2CaTi_{0.875}Nb_{0.1}O_3$	4.37	30
$0.7(0.75Na_{0.5}Bi_{0.5}TiO_3$-$0.25SrTiO_3)$-$0.3CaTi_{0.875}Nb_{0.1}O_3$	5.29	37
$0.6(0.75Na_{0.5}Bi_{0.5}TiO_3$-$0.25SrTiO_3)$-$0.4CaTi_{0.875}Nb_{0.1}O_3$	7.13	42
$0.5(0.75Na_{0.5}Bi_{0.5}TiO_3$-$0.25SrTiO_3)$-$0.5CaTi_{0.875}Nb_{0.1}O_3$	6.49	45
$0.88Bi_{0.5}Na_{0.5}TiO_3$-$0.12BaTiO_3$	0.23	10
$0.78Bi_{0.5}Na_{0.5}TiO_3$-$0.12BaTiO_3$-$0.1NaNbO_3$	0.88	10
$0.68Bi_{0.5}Na_{0.5}TiO_3$-$0.12BaTiO_3$-$0.2NaNbO_3$	0.83	10
$0.63Bi_{0.5}Na_{0.5}TiO_3$-$0.12BaTiO_3$-$0.25NaNbO_3$	0.74	10

$0.58Bi_{0.5}Na_{0.5}TiO_3$-$0.12BaTiO_3$-$0.3NaNbO_3$	0.57	10
$[(Bi_{0.5}Na_{0.5})_{0.94}Ba_{0.06}]_{0.94}La_{0.04}TiO_3$	1.99	21
$[(Bi_{0.5}Na_{0.5})_{0.94}Ba_{0.06}]_{0.88}La_{0.08}TiO_3$	3.52	25
$[(Bi_{0.5}Na_{0.5})_{0.94}Ba_{0.06}]_{0.85}La_{0.1}TiO_3$	5.03	36
$[(Bi_{0.5}Na_{0.5})_{0.94}Ba_{0.06}]_{0.82}La_{0.12}TiO_3$	5.89	43
$Bi_{0.5}Na_{0.5}TiO_3$	0.36	12
$0.75Bi_{0.5}Na_{0.5}TiO_3$-$0.25SrTiO_3$	1.14	19
$0.65Bi_{0.5}Na_{0.5}TiO_3$-$0.35SrTiO_3$	1.76	2.6
$0.88(0.65Bi_{0.5}Na_{0.5}TiO_3$-$0.35SrTiO_3)$-$0.12Bi(Mg_{0.5}Hf_{0.5})O_3$	5.59	39
$(Bi_{0.5}Na_{0.5})_{0.65}Sr_{0.35}TiO_3$	2.23	22
$0.95(Bi_{0.5}Na_{0.5})_{0.65}Sr_{0.35}TiO_3$-$0.05BiMg_{0.5}Sn_{0.5}O_3$	3.12	22
$0.9(Bi_{0.5}Na_{0.5})_{0.65}Sr_{0.35}TiO_3$-$0.1BiMg_{0.5}Sn_{0.5}O_3$	3.24	22
$0.85(Bi_{0.5}Na_{0.5})_{0.65}Sr_{0.35}TiO_3$-$0.15BiMg_{0.5}Sn_{0.5}O_3$	2.02	22
$(Bi_{0.5}Na_{0.5})_{0.7}Sr_{0.3}TiO_3$	1.44	14
$0.95(Bi_{0.5}Na_{0.5})_{0.7}Sr_{0.3}TiO_3$-$0.05Bi(Mg_{0.5}Sn_{0.5})O_3$	1.59	15
$0.9(Bi_{0.5}Na_{0.5})_{0.7}Sr_{0.3}TiO_3$-$0.1Bi(Mg_{0.5}Sn_{0.5})O_3$	2.13	22
$0.85(Bi_{0.5}Na_{0.5})_{0.7}Sr_{0.3}TiO_3$-$0.15Bi(Mg_{0.5}Sn_{0.5})O_3$	3.73	28
$0.8(Bi_{0.5}Na_{0.5})_{0.7}Sr_{0.3}TiO_3$-$0.2Bi(Mg_{0.5}Sn_{0.5})O_3$	1.81	20
$0.94Na_{0.5}Bi_{0.5}TiO_3$-$0.06BaTiO_3$	0.66	12
$0.82(0.94Na_{0.5}Bi_{0.5}TiO_3$-$0.06BaTiO_3)$-$0.18CaTi_{0.75}Ta_{0.2}O_3$	2.47	19
$0.79(0.94Na_{0.5}Bi_{0.5}TiO_3$-$0.06BaTiO_3)$-$0.21CaTi_{0.75}Ta_{0.2}O_3$	6.11	30
$0.76(0.94Na_{0.5}Bi_{0.5}TiO_3$-$0.06BaTiO_3)$-$0.24CaTi_{0.75}Ta_{0.2}O_3$	9.55	41
$0.7Bi_{0.5}Na_{0.5}TiO_3$-$0.3Na_{0.91}Bi_{0.09}Nb_{0.94}Mg_{0.06}O_3$	5.5	37
$0.7Bi_{0.5}Na_{0.5}TiO_3$-$0.3Na_{0.91}Bi_{0.09}Nb_{0.94}Mg_{0.06}O_3$-$0.05CaZrO_3$	7.5	51
$0.7Bi_{0.5}Na_{0.5}TiO_3$-$0.3Na_{0.91}Bi_{0.09}Nb_{0.94}Mg_{0.06}O_3$-$0.1CaZrO_3$	4.8	48
$0.7Bi_{0.5}Na_{0.5}TiO_3$-$0.3Na_{0.91}Bi_{0.09}Nb_{0.94}Mg_{0.06}O_3$-$0.2CaZrO_3$	3.1	44
$0.9Bi_{0.395}Na_{0.325}Sr_{0.245}TiO_3$-$0.1Ca_{0.7}La_{0.2}TiO_3$	2.87	32
$0.85Bi_{0.395}Na_{0.32}5Sr_{0.245}TiO_3$-$0.15Ca_{0.7}La_{0.2}TiO_3$	5.31	52
$0.8Bi_{0.395}Na_{0.32}5Sr_{0.245}TiO_3$-$0.2Ca_{0.7}La_{0.2}TiO_3$	8.24	66
$0.75Bi_{0.395}Na_{0.32}5Sr_{0.245}TiO_3$-$0.25Ca_{0.7}La_{0.2}TiO_3$	7.94	76
$0.9Bi_{0.5}Na_{0.5}TiO_3$–$0.1Ba_{0.7}Sr_{0.3}Zr_{0.8}Sn_{0.2}O_3$	4.88	27
$0.85Bi_{0.5}Na_{0.5}TiO_3$–$0.15Ba_{0.7}Sr_{0.3}Zr_{0.8}Sn_{0.2}O_3$	6.29	34
$0.80Bi_{0.5}Na_{0.5}TiO_3$–$0.20Ba_{0.7}Sr_{0.3}Zr_{0.8}Sn_{0.2}O_3$	7.4	40
$0.75Bi_{0.5}Na_{0.5}TiO_3$–$0.25Ba_{0.7}Sr_{0.3}Zr_{0.8}Sn_{0.2}O_3$	6.57	46
$0.9Bi_{0.5}Na_{0.5}TiO_3$-$0.1Sr_{0.7}Bi_{0.2}Ti_{0.8}Hf_{0.2}O_3$	2.14	25
$0.8Bi_{0.5}Na_{0.5}TiO_3$-$0.2Sr_{0.7}Bi_{0.2}Ti_{0.8}Hf_{0.2}O_3$	4.07	27
$0.7Bi_{0.5}Na_{0.5}TiO_3$-$0.3Sr_{0.7}Bi_{0.2}Ti_{0.8}Hf_{0.2}O_3$	5.37	30
$0.6Bi_{0.5}Na_{0.5}TiO_3$-$0.4Sr_{0.7}Bi_{0.2}Ti_{0.8}Hf_{0.2}O_3$	5.48	41
$0.925Bi_{0.5}Na_{0.5}TiO_3$-$0.075AgNb_{0.5}Ta_{0.5}O_3$	2.28	32
$0.9Bi_{0.5}Na_{0.5}TiO_3$-$0.1AgNb_{0.5}Ta_{0.5}O_3$	4.07	36
$0.875Bi_{0.5}Na_{0.5}TiO_3$-$0.125AgNb_{0.5}Ta_{0.5}O_3$	5.10	36
$0.85Bi_{0.5}Na_{0.5}TiO_3$-$0.15AgNb_{0.5}Ta_{0.5}O_3$	5.12	40
$0.6Na_{0.5}Bi_{0.5}TiO_3$-$0.4Sr_{0.7}Bi_{0.2}TiO_3$	3.51	23
$0.98(0.6Na_{0.5}Bi_{0.5}TiO_3$-$0.4Sr_{0.7}Bi_{0.2}TiO_3)$-$0.02Ba(Mg_{1/3}Ta_{2/3})O_3$	5.31	36

$0.96(0.6Na_{0.5}Bi_{0.5}TiO_3\text{-}0.4Sr_{0.7}Bi_{0.2}TiO_3)\text{-}0.04Ba(Mg_{1/3}Ta_{2/3})O_3$	6.93	47
$0.94(0.6Na_{0.5}Bi_{0.5}TiO_3\text{-}0.4Sr_{0.7}Bi_{0.2}TiO_3)\text{-}0.06Ba(Mg_{1/3}Ta_{2/3})O_3$	7.79	52
$0.92(0.6Na_{0.5}Bi_{0.5}TiO_3\text{-}0.4Sr_{0.7}Bi_{0.2}TiO_3)\text{-}0.08Ba(Mg_{1/3}Ta_{2/3})O_3$	8.57	56
$0.9(0.6Na_{0.5}Bi_{0.5}TiO_3\text{-}0.4Sr_{0.7}Bi_{0.2}TiO_3)\text{-}0.1Ba(Mg_{1/3}Ta_{2/3})O_3$	8.24	57
$0.9Bi_{0.5}Na_{0.5}TiO_3\text{-}0.1Ba_{0.85}Ca_{0.15}Zr_{0.1}Ti_{0.9}O_3$	1.14	12
$0.95(0.9Bi_{0.5}Na_{0.5}TiO_3\text{-}0.1Ba_{0.85}Ca_{0.15}Zr_{0.1}Ti_{0.9}O_3)\text{-}0.05BaSnO_3$	4.97	37
$0.9(0.9Bi_{0.5}Na_{0.5}TiO_3\text{-}0.1Ba_{0.85}Ca_{0.15}Zr_{0.1}Ti_{0.9}O_3)\text{-}0.1BaSnO_3$	3.12	25
$0.85(0.9Bi_{0.5}Na_{0.5}TiO_3\text{-}0.1Ba_{0.85}Ca_{0.15}Zr_{0.1}Ti_{0.9}O_3)\text{-}0.15BaSnO_3$	3.21	27
$0.94Bi_{0.47}Na_{0.47}Ba_{0.06}TiO_3\text{-}0.06CaHfO_3$	2.64	19
$0.7(0.94Bi_{0.47}Na_{0.47}Ba_{0.06}TiO_3\text{-}0.06CaHfO_3)\text{-}0.3Bi_{0.2}Sr_{0.7}TiO_3$	4.12	25
$0.65(0.94Bi_{0.47}Na_{0.47}Ba_{0.06}TiO_3\text{-}0.06CaHfO_3)\text{-}0.35Bi_{0.2}Sr_{0.7}TiO_3$	5.11	33
$0.6(0.94Bi_{0.47}Na_{0.47}Ba_{0.06}TiO_3\text{-}0.06CaHfO_3)\text{-}0.4Bi_{0.2}Sr_{0.7}TiO_3$	6.19	41
$0.55(0.94Bi_{0.47}Na_{0.47}Ba_{0.06}TiO_3\text{-}0.06CaHfO_3)\text{-}0.45Bi_{0.2}Sr_{0.7}TiO_3$	6.01	45
$(Bi_{0.5}Na_{0.5})_{0.7}Sr_{0.3}Ti_{0.98}(Fe_{0.5}Nb_{0.5})_{0.02}O_3$	1.54	12
$(Bi_{0.5}Na_{0.5})_{0.65}Sr_{0.35}Ti_{0.98}(Fe_{0.5}Nb_{0.5})_{0.02}O_3$	1.69	12
$(Bi_{0.5}Na_{0.5})_{0.6}Sr_{0.4}Ti_{0.98}(Fe_{0.5}Nb_{0.5})_{0.02}O_3$	2.72	17
$(Bi_{0.5}Na_{0.5})_{0.55}Sr_{0.45}Ti_{0.98}(Fe_{0.5}Nb_{0.5})_{0.02}O_3$	1.53	12
$0.6Bi_{0.5}Na_{0.5}TiO_3\text{-}0.4Sr_{0.7}Bi_{0.2}TiO_3$	1.97	17
$0.97(0.6Bi_{0.5}Na_{0.5}TiO_3\text{-}0.4Sr_{0.7}Bi_{0.2}TiO_3)\text{-}0.03AgNbO_3$	2.92	21
$0.95(0.6Bi_{0.5}Na_{0.5}TiO_3\text{-}0.4Sr_{0.7}Bi_{0.2}TiO_3)\text{-}0.05AgNbO_3$	3.62	25
$0.93(0.6Bi_{0.5}Na_{0.5}TiO_3\text{-}0.4Sr_{0.7}Bi_{0.2}TiO_3)\text{-}0.07AgNbO_3$	3.33	27
$0.94Na_{0.5}Bi_{0.5}TiO_3\text{-}0.06BaTiO_3$	0.94	16
$0.95(0.94Na_{0.5}Bi_{0.5}TiO_3\text{-}0.06BaTiO_3)\text{-}0.05CaTi_{0.8}Hf_{0.2}O_3$	4.28	24
$0.85(0.94Na_{0.5}Bi_{0.5}TiO_3\text{-}0.06BaTiO_3)\text{-}0.15CaTi_{0.8}Hf_{0.2}O_3$	5.23	33
$0.75(0.94Na_{0.5}Bi_{0.5}TiO_3\text{-}0.06BaTiO_3)\text{-}0.25CaTi_{0.8}Hf_{0.2}O_3$	8.92	41
$0.7(0.94Na_{0.5}Bi_{0.5}TiO_3\text{-}0.06BaTiO_3)\text{-}0.3CaTi_{0.8}Hf_{0.2}O_3$	6.51	43
$0.6Bi_{0.5}Na_{0.5}TiO_3\text{-}0.4Sr_{0.7}Bi_{0.2}TiO_3$	3.64	26
$0.96(0.6Bi_{0.5}Na_{0.5}TiO_3\text{-}0.4Sr_{0.7}Bi_{0.2}TiO_3)\text{-}0.04La(Mg_{0.5}Ti_{0.5})O_3$	6.0	34
$0.92(0.6Bi_{0.5}Na_{0.5}TiO_3\text{-}0.4Sr_{0.7}Bi_{0.2}TiO_3)\text{-}0.08La(Mg_{0.5}Ti_{0.5})O_3$	6.79	39
$0.88(0.6Bi_{0.5}Na_{0.5}TiO_3\text{-}0.4Sr_{0.7}Bi_{0.2}TiO_3)\text{-}0.12La(Mg_{0.5}Ti_{0.5})O_3$	5.94	41
$Bi_{0.5}Na_{0.5}TiO_3$	0.83	20
$0.9Bi_{0.5}Na_{0.5}TiO_3\text{-}0.1BaFe_{0.5}Nb_{0.5}O_3$	1.01	20
$0.8Bi_{0.5}Na_{0.5}TiO_3\text{-}0.2BaFe_{0.5}Nb_{0.5}O_3$	2.01	24
$0.7Bi_{0.5}Na_{0.5}TiO_3\text{-}0.3BaFe_{0.5}Nb_{0.5}O_3$	4.39	34
$0.6Bi_{0.5}Na_{0.5}TiO_3\text{-}0.4BaFe_{0.5}Nb_{0.5}O_3$	7.99	50
$0.5Bi_{0.5}Na_{0.5}TiO_3\text{-}0.5BaFe_{0.5}Nb_{0.5}O_3$	2.21	30
$Bi_{0.5}(Na_{0.9}Li_{0.1})_{0.5}TiO_3$	2.22	33

Supplementary Table 2. **The descriptor of machine learning.** Abbreviation and description for the full descriptor of the element.

Feature	Description
**RSC12**	Shannon's (1976) ionic radii (12-coordination)
**RSC6**	Shannon's (1976) ionic radii (6-coordination)
**RC**	Covalent radii
**RP**	Pseudopotential core radii
**Rdce**	Core electron distance (Schubert)
**Rdve**	Valence electron distance (Schubert)
**VEC_Z**	Ratio of valence electron number to nuclear charge
**Valence**	Valence
**AN**	Atomic number
**AV**	Atomic volume
**AW**	Atomic weight
**AR**	Atomic radius
**RCov-c**	Covalent radii (Cordero)
**DP**	Atomic dipole polarizability
**EN_MB**	Electronegativity (Matyonov-Batsanov)
**Electrons**	Electron number
**EN-A**	Allen electronegativity
**EN-G**	Ghosh electronegativity
**EN-P**	Pauling electronegativity
**EA**	Electron affinity
**HOF**	Heat of formation
**MN**	Mendeleev number
**RM**	Single bond metallic radius
**RMC12**	12 single bond metallic radii of immediately neighboring atoms
**Neutrons**	Number of Neutrons
**Period**	Periods in the periodic table of the elements
**RVdw-A**	Alvarez's van der Waals radius
**RVdw**	Van der Waals radius
**I1**	First ionization energy
**I2**	Second ionization energy
**I3**	Third ionization energy
**OF**	Octahedral factor calculated by Shannon's ionic radii

Supplementary Table 3. The selected descriptors. The strong correlation check and recursive feature elimination results in remaining descriptors.

	After the checking for strong correlations (35 features)			After the recursive feature elimination (11 features)
**Features**	RSC12(A)	En-P(A)	Vec_Z(B)	RSC12(A)
	RC(A)	EA(A)	Valence(B)	Rdve(A)
	Rdce(A)	HOF(A)	AN(B)	En_MB(A)
	Rdve(A)	MN(A)	DP(B)	EA(A)
	Vec_Z(A)	Period(A)	En_MB(B)	Period(A)
	Valence(A)	I1(A)	En-A(B)	I2(A)
	AN(A)	I2(A)	En-G(B)	RSC6(B)
	AV(A)	I3(A)	MN(B)	En_MB(B)
	AR(A)	RSC6(B)	Period(B)	En-A(B)
	DP(A)	RP(B)	I3(B)	En-G(B)
	En_MB(A)	Rdce(B)	Electric field	Electric field
	En-G(A)	Rdve(B)		

Supplementary Table 4. The hyperparameters of Random Forest in machine learning. Random Forest training results and the used hyperparameters.

	n_estimators	max_depth	random_state	min_samples_split	min_samples_leaf	R^2 (test)	R^2 (train)
RF	200	8	None	2	1	0.84	0.97

Supplementary Table 5. The hyperparameters of Gradient Boosted Regressor in machine learning. Gradient Boosted Regressor training results and the used hyperparameters.

	loss	n_estimators	max_depth	learning_rate	min_samples_split	R^2 (test)	R^2 (train)
GBR	squared_error	200	8	0.0115	2	0.74	0.98

Supplementary Table 6. The hyperparameters of Decision Tree Regressor in machine learning. Decision Tree Regressor training results and the used hyperparameters.

	max_depth	random_state	min_samples_split	R^2 (test)	R^2 (train)
DTR	8	None	4	0.66	0.98

Supplementary Table 7. **The hyperparameters of Radial Basis Function Kernel Support Vector Regression in machine learning**. Radial Basis Function Kernel Support Vector Regression training results and the used hyperparameters.

	kernel	C	gamma	R^2 (test)	R^2 (train)
SVR.rbf	rbf	1000	0.0075	0.47	0.99

Supplementary Table 8. **The hyperparameters of Bayesian Ridge Regression in machine learning**. Bayesian Ridge Regression training results and the used hyperparameters.

	tol	fit_intercept	max_iter	compute_score	R^2 (test)	R^2 (train)
BR	1e-5	False	300	True	0.73	0.84

Supplementary Table 9. **Comparison of the energy-storage properties.** The W_{rec}, η, and E_B between sample A and reported lead-free bulk ceramics.

System	W_{rec} (J cm^{-3})	η (%)	E_B (kV mm^{-1})	Ref.
BNT-based	0.684	87.5	12.9	[1]
	0.71	67	7	[2]
	0.8	65	7	[3]
	0.85	67	10	[4]
	0.87	82.1	9.4	[5]
	0.92	70	9.6	[6]
	1.03	72.7	14	[7]
	1.17	82.4	10.4	[8]
	1.2	76.9	11	[9]
	1.23	74.7	9	[10]
	1.32	76.7	9.5	[11]
	1.4	82.3	13	[12]
	1.56	92.5	12	[13]
	1.62	79.5	19	[14]
	1.7	87.2	13	[15]
	1.72	85.6	10.5	[16]
	1.83	67	12	[17]
	1.83	82.3	18.5	[18]
	1.91	86.4	19	[19]
	2.03	62	12	[20]
	2.04	82.4	13.5	[21]
	2.08	88.8	24.5	[22]
	2.1	92.9	17	[23]
	2.12	83	18	[24]
	2.13	67.8	21.5	[25]
	2.2	75	16	[26]
	2.2	72.4	23	[27]
	2.32	80.1	25	[28]
	2.41	81.6	24	[29]
	2.42	64.5	14.3	[30]
	2.49	85	17	[31]
	2.72	81	17	[32]

	2.86	90.3	18	33
	2.9	80	33	34
	2.93	72	21	35
	3.02	75	21	36
	3.08	81.4	22	37
	3.09	77	18.2	38
	3.1	91	28	39
	3.18	60	21	40
	3.2	93	28	41
	3.4	90	31	42
	3.45	88	25	43
	3.72	90.7	29	44
	3.75	84.8	38	45
	3.97	81	25	46
	4.06	87.3	35	47
	4.1	83.2	25	48
	4.14	92.2	31.5	49
	4.18	83.6	27	50
	4.21	77.8	38	51
	5.55	85	35	52
	5.63	94	53.5	53
	6.19	93.5	41	54
	6.3	79.6	42	55
	6.43	88	50.5	56
	6.57	70	38	57
	7.02	85	39	58
	7.05	65	38.7	59
	7.3	80	54	60
	7.5	92	47	61
	7.6	90	41	62
	8	80	50	63
	8.33	90.8	55.5	64
	8.4	94	47.5	65
	8.91	78.4	41	66
	10.7	89	64	67
	13.6	94	66	68

	13.8	82.4	64	69
	15.1	82.4	64	70
	15.2	91	73	71
	16.21	90.5	80	72
	6.79	93	38.6	73
	10.57	87.8	55	74
	4.97	84.4	36.5	75
	8.58	93.5	56.4	76
	7.4	88.6	40	77
	8.24	80.3	66	78
	9.55	88	41	79
	5.88	77.7	43	80
	7.13	90.2	42.3	81
	8.46	85.9	52.5	82
	9.22	96.3	53.5	83
	0.6	80	10	84
	2.08	68	18	85
	2.17	83.9	24.5	86
	2.31	77.7	19	87
	2.88	76.9	15	88
	3.07	88	26	89
BKT-based	3.14	83.7	23	90
	4.25	87	36	91
	5.21	90.87	36	92
	6.34	93.32	42	93
	6.52	70	42.5	94
	7.57	81.4	46	95
	17.3	88.5	78	96
	5.33	75	29	97
	0.43	86	10.5	98
	0.59	72.8	16	99
	0.71	82.6	9.3	100
BT-based	0.86	94	11.5	101
	1.13	95.8	14	102
	1.15	92	28.7	103
	1.25	95	18.5	104

	1.36	74.3	15.2	105
	1.61	94.3	23	106
	1.62	99.8	22.5	107
	1.7	90	21	108
	1.75	85	23.5	109
	1.81	88	22.4	110
	1.81	81.9	15	111
	1.89	83	24	112
	2.01	88.6	30	113
	2.02	90.1	20.6	114
	2.03	94.5	30	115
	2.09	95.9	20	116
	2.21	91.6	28	117
	2.25	94	24	118
	2.47	94.4	25	119
	3.282	93	39.5	120
	2.53	86.6	20	121
	2.53	93.9	24	122
	2.9	86.8	30.1	123
	3	93.8	31.3	124
	3.2	91.5	33	125
	3.22	91.2	24.1	126
	3.27	90.22	48	127
	3.28	93	35.6	128
	3.34	85.7	40	129
	3.38	87	24	130
	3.54	75.6	48.4	131
	3.86	81.6	22	132
	4.03	96.2	37	133
	4.2	75.9	46.8	134
	4.23	93.4	41.8	135
	4.49	93	34	136
	4.55	81.8	45	137
	4.55	90	52	138
	4.6	90	43	139
	5.36	82.2	43.6	140

	5.92	81.7	55.2	141
	7.12	90	72	142
	8.2	92.2	59	143
	9.03	95.2	72	144
	9.04	87.2	54	145
	10.2	91.4	76.2	146
	10.28	97.11	56	147
	11.6	96.1	58	148
NN-based	0.55	63	13	149
	1.5	68.2	17.5	150
	1.6	50	20.7	151
	2.1	76	20	152
	2.2	62.7	25	153
	2.31	80.2	25.5	154
	2.8	82	30	155
	3.02	80.7	31	156
	3.14	84.5	34	157
	3.31	80.9	44	158
	3.41	90.8	28	159
	3.51	87	51	160
	3.51	80.1	35	161
	4.03	85.4	25	162
	4.5	90.3	28.8	163
	4.9	88	43	164
	4.9	72.6	50	165
	5	68	55	166
	5.01	86.1	62.7	167
	5.29	82.1	38	168
	5.53	82	57.5	169
	5.57	71	48	170
	6.43	82	53	171
	6.5	65.9	55	172
	6.5	94	45	173
	7.1	90	64.6	174
	7.33	83.68	53	175
	7.59	81.3	64	176

	8	90.4	80	177
	8.73	80.1	48	178
	10.59	87.6	55	179
	10.9	83	78.3	180
	12.2	69	68	181
	14.5	83.9	93	182
	16.2	82.3	97	183
	16.5	83.3	98.3	184
	18.5	78.7	91	185
	11.2	90.5	100	186
AN-based	2	46	15	187
	2.3	46	18	188
	2.5	57	15	189
	2.4	54	17.5	190
	2.6	86	20	191
	2.9	56	19	192
	3.12	63	23	193
	3.2	52	21	194
	3.3	50	20	195
	3.36	58.3	22	196
	3.4	62	22	197
	3.5	53.1	21	198
	3.65	84.31	21.5	199
	3.7	57.5	23.5	200
	4.2	69	23.3	201
	4.4	73	27.3	202
	4.5	63	30	203
	4.5	64	35	204
	4.6	57.5	22	205
	4.6	62.5	39.4	206
	4.8	68	26.1	207
	4.87	63.5	28.5	208
	5.2	68.5	29	209
	6.3	90	47	210
	6.5	71	37	211
	6.9	61	49	212

	7.01	77	54.12	213
BF-based	1.56	75	12.5	214
	1.66	82	13	215
	1.75	81	15.5	216
	1.82	44.4	18	217
	1.97	81.7	5	218
	2.1	53	18	219
	2.1122	84	19.5	220
	2.4	90.4	18	221
	2.56	71	16	222
	2.8	55.8	20	223
	3.08	85.6	23	224
	3.2	73.7	29	225
	3.38	59	23	226
	3.64	74	28	227
	3.9	80	25	228
	3.9	80	27.1	228
	5.2	88	35.4	229
	7.4	81	68	230
	8.12	90	36	231
	8.2	74.1	46	232
	13.9	89.6	63	233
	15.9	87.7	68	234
KNN-based	1.5	50	18	235
	2	60.5	20.6	236
	2.02	81.4	29.5	237
	3.39	51.7	33	238
	3.5	86.6	32.6	239
	3.6	74.2	34	240
	4.03	52	40	241
	4.08	62.7	30	242
	4.85	88.2	48	243
	4.87	53.3	22	244
	6.7	92	60	245
	7.4	78	80	246
	8.09	88.46	87	247

	10.06	90.8	74	248
	13.1	88	74	249
ST-based	1.4	90	19.6	250
	1.45	86	16	251
	1.7	67.9	21	252
	1.78	77.1	17	253
	1.89	77	19	254
	2.05	85	39	255
	2.06	95	33	256
	2.1	97.6	29	257
	2.35	65	18	258
	2.35	77	42.2	259
	2.59	85	32.3	260
	2.83	85	32	261
	2.84	71.5	28.5	262
	3.1	93	36	263
	3.37	94.9	44	264
	4	89.5	51	265
	4.2	88	38	266
	5.21	91.55	54.3	267
	6	92	44	268
	7.3	86.3	50.4	269
	8.4	90	75	270
Non-perovskite -based	11	81.9	75.3	271
	8.9	93	70	272
	9	84	66	273

Supplementary Table 10. **Comparison of total electrical resistivity**. The total electrical resistivity at around 500~550 °C between sample A and some representative lead-free bulk energy-storage ceramics.

System	Z' (MΩ·cm)	Ref.
BT-based	0.1	96
	0.65	138
BNKT-based	0.48	96
NN-based	0.5	177
BNT-based	0.055	147
	0.6	70
	1.25	71
KNN-based	2.17	274

Supplementary Table 11. **Comparison of the energy-storage properties.** Comparison of the W_{rec} between high-entropy sample A and other reported high-entropy energy-storage bulk ceramics.

W_{rec} (J cm^{-3})	Ref.
1.02	275
1.07	276
1.094	277
1.12	276
1.32	278
1.37	279
1.51	280
1.56	281
2.24	282
2.43	283
2.47	284
2.53	285
2.61	286
2.72	287
2.95	288
3.37	289
3.4	290
3.51	291
3.52	292
3.85	293
3.86	294

4.46	295
4.89	296
4.9	297
5.18	298
5.58	299
5.6	300
6.21	301
6.44	302
6.66	285
6.89	303
7.2	304
7.3	305
7.6	306
8.11	307
8.2	143
8.71	308
8.8	309
8.9	272
9.8	310
10.06	248
10.6	311
10.7	67
11.0	271
11.6	148
13.8	312

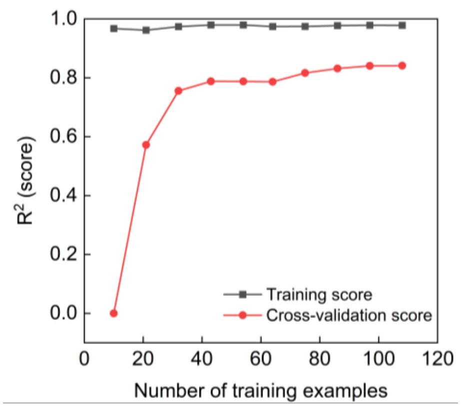

Supplementary Fig. 1 Learning Curves of machine learning. Learning Curves for Random Forest regression model on the dataset.

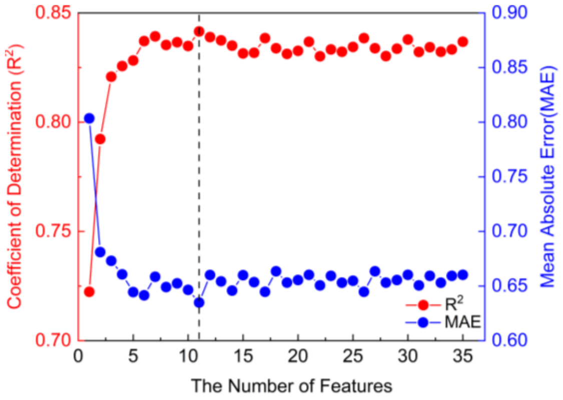

Supplementary Fig. 2 Feature selection. Recursive feature elimination cross-validation via random forest modeling based on a subset of 35 reserved descriptors.

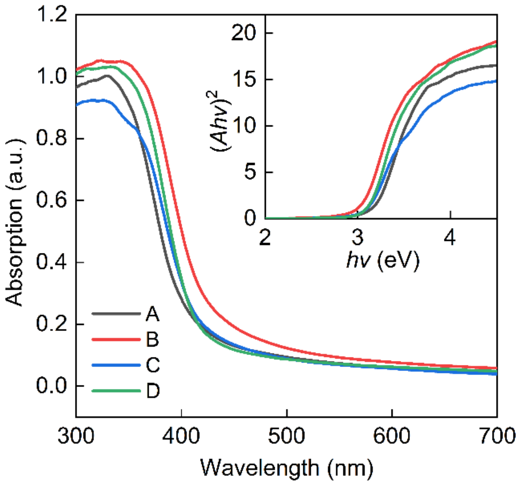

Supplementary Fig. 3 Bandgap of samples. UV-Vis absorption spectra of samples A, B, C and D. The inset represents the calculated bandgap (E_g).

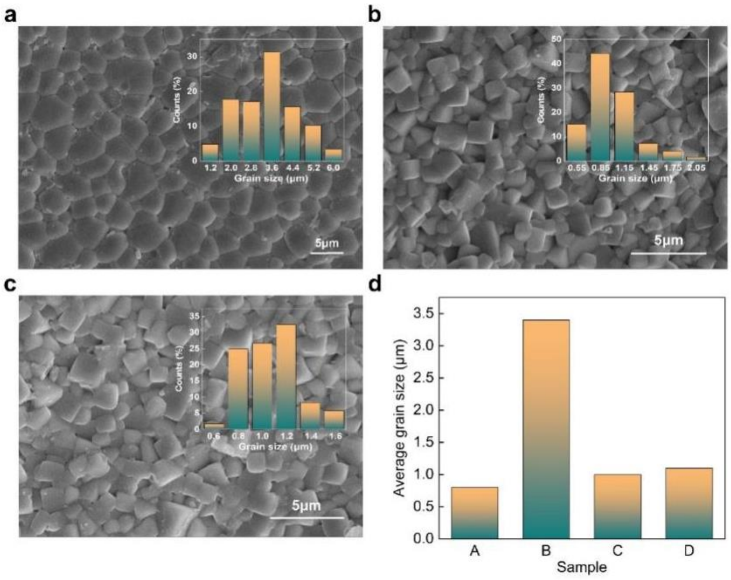

Supplementary Fig. 4 Scanning electron microscopy (SEM) images of the samples. SEM images displaying the surface morphology and grain size distribution of sample **a** B, **b** C, **c** D and **d** Comparison of average grain size of four samples.

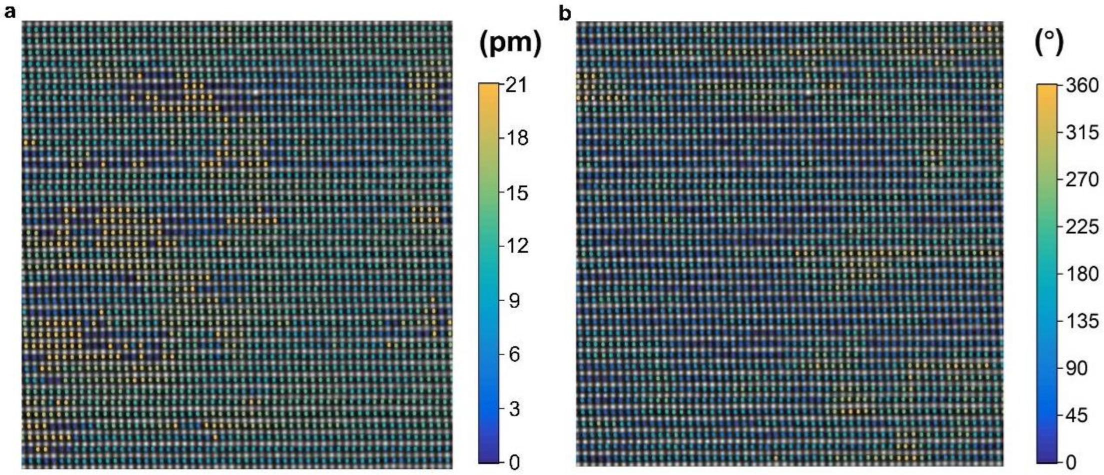

Supplementary Fig. 5 Atomic displacement magnitude and angle mapping along [110]$_{c}$. a Magnitudes and **b** Angles mapping of atomic displacement vectors of sample A.

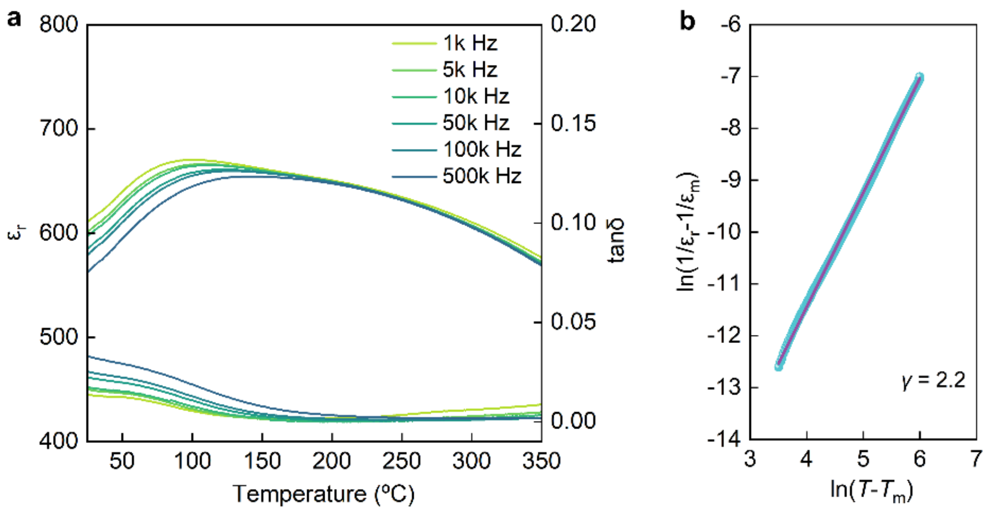

Supplementary Fig. 6 Dielectric spectrum of sample A. a Temperature-dependent dielectric spectrum, and **b** Modified Curie-Weiss distribution.

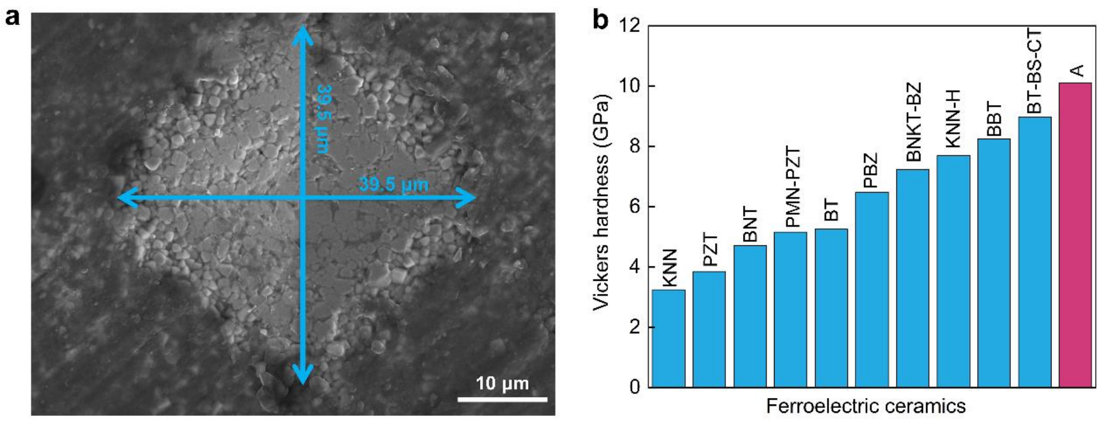

Supplementary Fig. 7 Vickers hardness of sample A. a Indentation of Vickers hardness tester bit on sample A ceramic observed by SEM. The Vickers hardness H_v is calculated to be about 10.1 GPa. **b** Comparison of H_v between sample A ceramic and some representative ferroelectric ceramics.

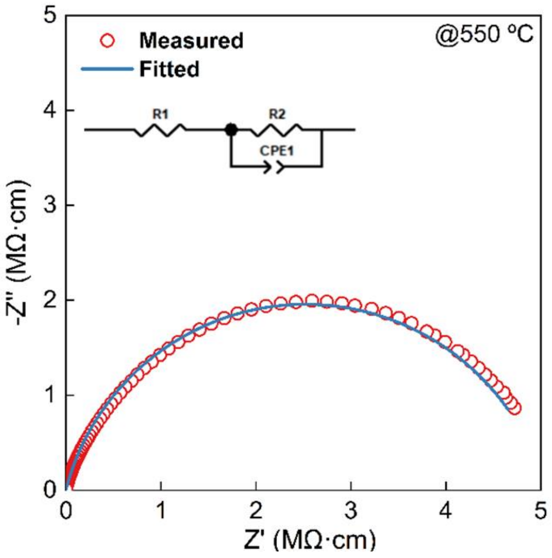

Supplementary Fig. 8 Complex impedance spectrum of sample A. Measured and fitted complex impedance spectrum of sample A.

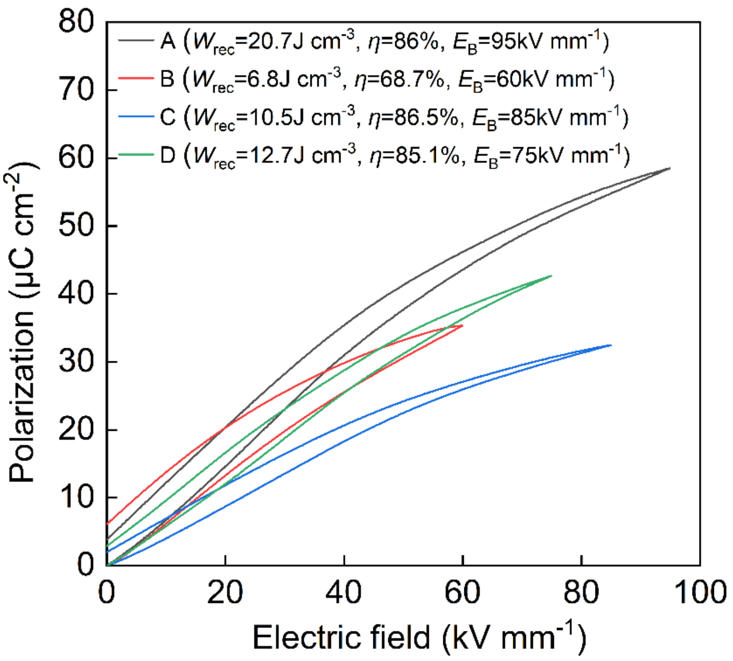

Supplementary Fig. 9 Evaluation of energy-storage properties. Unipolar *P*-*E* loops measured under breakdown field and calculated energy-storage properties of sample A, B, C and D.

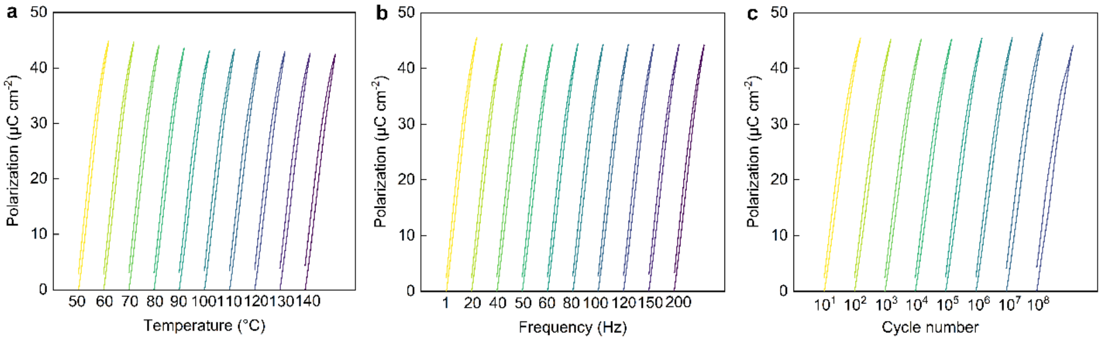

Supplementary Fig. 10 Stability of the energy storage properties of sample A under an electric field of 60 kV mm^{-1}. **a** Temperature dependent, **b** Frequency dependent, **c** Cycling dependent *P*-*E* loops.

Supplementary References

1. Liu, J., Ren, K., Ma, C., Du, H. & Wang, Y. Dielectric and energy storage properties of flash-sintered high-entropy $(Bi_{0.2}Na_{0.2}K_{0.2}Ba_{0.2}Ca_{0.2})TiO_3$ ceramic. *Ceram. Int.* **46**, 20576–20581 (2020).
2. Xu, Q. *et al.* Enhanced energy storage properties of $NaNbO_3$ modified $Bi_{0.5}Na_{0.5}TiO_3$ based ceramics. *J. Eur. Ceram. Soc.* **35**, 545–553 (2015).
3. Xie, H. *et al.* Comparative studies on structure, dielectric, strain and energy storage properties of $(Bi_{0.5}Na_{0.5})_{0.94}Ba_{0.06}Ti_{0.965}(Mg_{1/3}Nb_{2/3})_{0.035}O_3$ lead-free ceramics prepared by traditional and two-step sintering method. *J. Mater. Sci. Mater. Electron.* (2018).
4. Yao, Y. *et al.* Enhanced dielectric and energy-storage properties in ZnO-doped $0.9(0.94Na_{0.5}Bi_{0.5}TiO_3{-}0.06BaTiO_3){-}0.1NaNbO_3$ ceramics. *Ceram. Int.* **44**, 5961–5966 (2018).
5. Enhanced energy storage properties of $(1{-}x)Bi_{0.5}Na_{0.5}TiO_3$-$xBa_{0.85}Ca_{0.15}Ti_{0.9}Zr_{0.1}O_3$ ceramics. *Mater. Lett.* **174**, 110–113 (2016).
6. Li, D. *et al.* Effect of $(Nb_{2/3}Mg_{1/3})^{4+}$ complex on the dielectric and ferroelectric properties of $(Ba_{0.3}Sr_{0.7})_{0.35}(Bi_{0.5}Na_{0.5})_{0.65}TiO_3$ ceramics for energy storage. *J. Mater. Sci. Mater. Electron.* **31**, 3648–3653 (2020).
7. Tao, C.-W. *et al.* $Bi_{0.5}Na_{0.5}TiO_3$-$BaTiO_3$-$K_{0.5}Na_{0.5}NbO_3$:ZnO relaxor ferroelectric composites with high breakdown electric field and large energy storage properties. *J. Eur. Ceram. Soc.* **38**, 4946–4952 (2018).
8. Luo, L., Wang, B., Jiang, X. & Li, W. Energy storage properties of (1-x)$(Bi_{0.5}Na_{0.5})TiO_3$-$xKNbO_3$ lead-free ceramics. *J. Mater. Sci.* **49**, 1659–1665 (2014).
9. Dong, G., Fan, H., Liu, H. & Jia, Y. Enhanced temperature stable dielectric property and energy-storage performance of (1-*x*)$(0.66Bi_{0.5}Na_{0.5}TiO_3{-}0.34Sr_{0.7}Bi_{0.2}TiO_3)$-$xK_{0.5}Nd_{0.5}TiO_3$ lead-free relaxor electroceramics. *Ceram. Int.* **46**, 23194–23199 (2020).
10. Yan, B. *et al.* Enhanced energy-storage performance and thermally stable permittivity for $K_{0.5}Na_{0.5}NbO_3$ modified $[(Na_{0.5}Bi_{0.5})_{0.84}Sr_{0.16}]_{0.98}La_{0.01}TiO_3$ lead-free perovskite ceramics. *Ceram. Int.* **46**, 9637–9645 (2020).
11. Yadav, A. K. *et al.* Enhanced storage energy density and fatigue free properties for

$0.94Bi_{0.50}(Na_{0.78}K_{0.22})_{0.50}Ti_{1-x}(Al0.50Nb0.50)_xO_{3-0.06}BaZrO_3$ ceramics. *Ceram. Int.* **46**, 17044–17052 (2020).

12. Wang, H. *et al.* An effective approach to achieve high energy storage density and efficiency in BNT-based ceramics by doping $AgNbO_3$. *Dalton Trans.* **48**, 17864–17873 (2019).

13. Sui, J. *et al.* Enhanced energy-storage performance and temperature-stable dielectric properties of $(1\text{-}x)[(Na_{0.5}Bi_{0.5})_{0.95}Ba_{0.05}]_{0.98}La_{0.02}TiO_{3}\text{-}xK_{0.5}Na_{0.5}NbO_3$ lead-free ceramics. *Ceram. Int.* **45**, 20427–20434 (2019).

14. Pu, Y., Yao, M., Zhang, L. & Chen, M. Enhanced energy storage density of $0.55Bi_{0.5}Na_{0.5}TiO_{3}\text{-}0.45Ba_{0.85}Ca_{0.15}Ti_{0.85}Zr_{0.1}Sn_{0.05}O_3$ with MgO addition. *J. Alloys Compd.* **702**, 171–177 (2017).

15. Wu, J. *et al.* Perovskite $Sr_x(Bi_{1-x}Na_{0.97-x}Li_{0.03})_{0.5}TiO_3$ ceramics with polar nano regions for high power energy storage. *Nano Energy* **50**, 723–732 (2018).

16. Hu, B., Fan, H., Ning, L., Wen, Y. & Wang, C. High energy storage performance of $[(Bi_{0.5}Na_{0.5})_{0.94}Ba_{0.06}]_{0.97}La_{0.03}Ti_{1-x}(Al_{0.5}Nb_{0.5})_xO_3$ ceramics with enhanced dielectric breakdown strength. *Ceram. Int.* **44**, 15160–15166 (2018).

17. Wang, C. *et al.* $[Bi_{0.5}(Na_{0.4-x}Li_xK_{0.1})]0.96Sr_{0.04}Ti_{0.975}Ta_{0.025}O_3$ lead-free relaxor ceramics with the enhanced recoverable energy density. *Ceram. Int.* **46**, 715–721 (2020).

18. Xie, J. *et al.* Enhanced energy storage properties of $Sr(Sc_{0.5}Nb_{0.5})O_3$ modified $(Bi_{0.47}La_{0.03}Na_{0.5})_{0.94}Ba_{0.06}TiO_3$ lead-free ceramics. *J. Mater. Sci.* **55**, 13578–13589 (2020).

19. Zhang, L., Pu, X., Chen, M., Bai, S. & Pu, Y. Influence of $BaSnO_3$ additive on the energy storage properties of $Na_{0.5}Bi_{0.5}TiO_3$-based relaxor ferroelectrics. *J. Eur. Ceram. Soc.* **38**, 2304–2311 (2018).

20. Li, J. *et al.* Enhanced energy storage performance under low electric field in Sm^{3+} doped $AgNbO_3$ ceramics. *J. Materiomics* **8**, 266–273 (2022).

21. Liu, Z. *et al.* Mediating the confliction of polarizability and breakdown electric-field strength in BNST relaxor ferroelectric for energy storage applications. *J. Alloys Compd.* **823**, 153772 (2020).

22. Zhang, L., Pu, Y. & Chen, M. Influence of $BaZrO_3$ additive on the energy-storage properties of $0.775Na_{0.5}Bi_{0.5}TiO_3\text{-}0.225BaSnO_3$ relaxor ferroelectrics. *J. Alloys Compd.* **775**, 342–347 (2019).

23. Wang, H. *et al.* Enhanced energy density and discharged efficiency of lead-free relaxor $(1\text{-}x)[(Bi_{0.5}Na_{0.5})_{0.94}Ba_{0.06}]_{0.98}La_{0.02}TiO_3\text{-}xKNb_{0.6}Ta_{0.4}O_3$ ceramic capacitors. *Chem. Eng. J.* **394**, 124879 (2020).

24. Li, T. *et al.* High energy storage density and efficiency with excellent temperature and frequency stabilities under low operating field achieved in $Ag_{0.91}Sm_{0.03}NbO_3$-modified $Na_{0.5}Bi_{0.5}TiO_3\text{-}BaTiO_3$ ceramics. *J. Mater. Sci. Mater. Electron.* **31**, 16928–16937 (2020).

25. Zhang, L., Pu, Y., Chen, M. & Liu, G. Antiferroelectric-like properties in MgO-modified $0.775Na_{0.5}Bi_{0.5}TiO_3\text{-}0.225BaSnO_3$ ceramics for high power energy storage. *J. Eur. Ceram. Soc.* **38**, 5388–5395 (2018).

26. Qiao, X. *et al.* Enhanced energy density and thermal stability in relaxor ferroelectric $Bi_{0.5}Na_{0.5}TiO_3\text{-}Sr_{0.7}Bi_{0.2}TiO_3$ ceramics. *J. Eur. Ceram. Soc.* (2019).

27. Zhang, F. *et al.* High energy storage density realized in $Bi_{0.5}Na_{0.5}TiO_3$-based relaxor ferroelectric ceramics at ultralow sintering temperature. *J. Eur. Ceram. Soc.* **41**, 368–375 (2021).

28. Yao, K. *et al.* $Bi_{0.5}Na_{0.5}TiO_3\text{–}Sr_{0.85}Bi_{0.1}TiO_3$ ceramics with high energy storage properties and extremely fast discharge speed via regulating relaxation temperature. *Ceram. Int.* **47**, 11294–11303 (2021).

29. Hu, D. *et al.* Significantly improved recoverable energy density and ultrafast discharge rate of $Na_{0.5}Bi_{0.5}TiO_3$-based ceramics. *Ceram. Int.* **46**, 15364–15371 (2020).

30. Yin, J., Zhang, Y., Lv, X. & Wu, J. Ultrahigh energy-storage potential under low electric field in bismuth sodium titanate-based perovskite ferroelectrics. *J. Mater. Chem. A* **6**, 9823–9832 (2018).
31. Bilal, M. K., Bashir, R., Asif, S. U., Wang, J. & Hu, W. Enhanced energy storage properties of $0.7Bi_{0.5}Na_{0.5}TiO_3$-$0.3SrTiO_3$ ceramic through the addition of $NaNbO_3$. *Ceram. Int.* **47**, 30922–30928 (2021).
32. Kang, R. *et al.* Energy storage performance of $Bi_{0.5}Na_{0.5}TiO_3$-based relaxor ferroelectric ceramics with superior temperature stability under low electric fields. *Chem. Eng. J.* **410**, 128376 (2021).
33. Kang, R. *et al.* Domain Engineered Lead-Free Ceramics with Large Energy Storage Density and Ultra-High Efficiency under Low Electric Fields. *ACS Appl. Mater. Interfaces* **13**, 25143–25152 (2021).
34. Wang, Y. *et al.* Superior energy storage performance in $Bi_{0.5}Na_{0.5}TiO_3$ based ceramics via synergistic design of multi-size domain construction and multiple phase structures. *Chem. Eng. J.* **500**, 156460 (2024).
35. Zhu, C. *et al.* High temperature lead-free BNT-based ceramics with stable energy storage and dielectric properties. *J. Mater. Chem. A* **8**, 683–692 (2020).
36. He, Z. *et al.* Low electric field induced high energy storage capability of the free-lead relaxor ferroelectric $0.94Bi_{0.5}Na_{0.5}TiO_3$-$0.06BaTiO_3$-based ceramics. *Ceram. Int.* **47**, 11611–11617 (2021).
37. Wu, Y. *et al.* Enhanced energy storage properties in sodium bismuth titanate-based ceramics for dielectric capacitor applications. *J. Mater. Chem. C* **7**, 6222–6230 (2019).
38. Wang, M. *et al.* Relaxor ferroelectric $Bi_{0.5}Na_{0.5}TiO_3$–$Sr_{0.7}Nd_{0.2}TiO_3$ ceramics with high energy storage density and excellent stability under a low electric field. *J. Phys. Chem. Solids* **157**, 110209 (2021).
39. Huang, Y. *et al.* $(Bi_{0.51}Na_{0.47})TiO_3$ based lead free ceramics with high energy density and efficiency. *J. Materiomics* **5**, 385–393 (2019).
40. Zhang, L., Pu, Y. & Chen, M. Ultra-high energy storage performance under low electric fields in $Na_{0.5}Bi_{0.5}TiO_3$-based relaxor ferroelectrics for pulse capacitor applications. *Ceram. Int.* **46**, 98–105 (2020).
41. Huang, Y., Guo, Q., Hao, H., Liu, H. & Zhang, S. Tailoring properties of $(Bi_{0.51}Na_{0.47})TiO_3$ based dielectrics for energy storage applications. *J. Eur. Ceram. Soc.* **39**, 4752–4760 (2019).
42. Yang, L., Kong, X., Cheng, Z. & Zhang, S. Ultra-high energy storage performance with mitigated polarization saturation in lead-free relaxors. *J. Mater. Chem. A* **7**, 8573–8580 (2019).
43. Lin, Y., Li, D., Zhang, M. & Yang, H. $(Na_{0.5}Bi_{0.5})_{0.7}Sr_{0.3}TiO_3$ modified by Bi(Mg2/3Nb1/3)O3 ceramics with high energy-storage properties and an ultrafast discharge rate. *J. Mater. Chem. C* **8**, 2258–2264 (2020).
44. Zhang, X. *et al.* Enhancement of recoverable energy density and efficiency of lead-free relaxor-ferroelectric BNT-based ceramics. *Chem. Eng. J.* **406**, 126818 (2021).
45. Shi, P. *et al.* $Bi_{0.5}Na_{0.5}TiO_3$-based lead-free ceramics with superior energy storage properties at high temperatures. *Compos. Part B Eng.* **215**, 108815 (2021).
46. Zheng, L. *et al.* Significantly tailored energy-storage performances in $Bi_{0.5}Na_{0.5}TiO_3$–$SrTiO_3$-based relaxor ferroelectric ceramics by introducing bismuth layer-structured relaxor $BaBi_2Nb_2O_9$ for capacitor application. *J. Mater. Chem. C* **9**, 5234–5243 (2021).
47. Hu, D. *et al.* Greatly enhanced discharge energy density and efficiency of novel relaxation ferroelectric BNT–BKT-based ceramics. *J. Mater. Chem. C* **8**, 591–601 (2020).
48. Yang, H., Tian, J., Lin, Y. & Ma, J. Realizing ultra-high energy storage density of lead-free $0.76Bi_{0.5}Na_{0.5}TiO_3$-$0.24SrTiO_3$-$Bi(Ni_{2/3}Nb_{1/3})O_3$ ceramics under low electric fields. *Chem. Eng. J.* **418**,

129337 (2021).
49. Qiao, X. *et al.* Superior comprehensive energy storage properties in $Bi_{0.5}Na_{0.5}TiO_3$-based relaxor ferroelectric ceramics. *Chem. Eng. J.* **388**, 124158 (2020).
50. Li, D. *et al.* A novel lead-free $Na_{0.5}Bi_{0.5}TiO_3$-based ceramic with superior comprehensive energy storage and discharge properties for dielectric capacitor applications. *J. Materiomics* **6**, 743–750 (2020).
51. Zhou, X. *et al.* Superior Thermal Stability of High Energy Density and Power Density in Domain-Engineered $Bi_{0.5}Na_{0.5}TiO_3$–$NaTaO_3$ Relaxor Ferroelectrics. *ACS Appl. Mater. Interfaces* **11**, 43107–43115 (2019).
52. Zhu, C. *et al.* Multiphase Engineered BNT-Based Ceramics with Simultaneous High Polarization and Superior Breakdown Strength for Energy Storage Applications. *ACS Appl. Mater. Interfaces* **13**, 28484–28492 (2021).
53. Yan, F. *et al.* Significantly enhanced energy storage density and efficiency of BNT-based perovskite ceramics via A-site defect engineering. *Energy Storage Mater.* **30**, 392–400 (2020).
54. Luo, C. *et al.* Significantly enhanced energy-storage properties of $Bi_{0.47}Na_{0.47}Ba_{0.06}TiO_3$-$CaHfO_3$ ceramics by introducing $Sr_{0.7}Bi_{0.2}TiO_3$ for pulse capacitor application. *Chem. Eng. J.* **429**, 132165 (2022).
55. Guo, B. *et al.* Energy storage performance of $Na_{0.5}Bi_{0.5}TiO_3$ based lead-free ferroelectric ceramics prepared via non-uniform phase structure modification and rolling process. *Chem. Eng. J.* **420**, 130475 (2021).
56. Wang, Z. *et al.* $(Bi_{0.5}Na_{0.5})TiO_3$-based relaxor ferroelectrics with medium permittivity featuring enhanced energy-storage density and excellent thermal stability. *Chem. Eng. J.* **427**, 131989 (2022).
57. Zhang, L. *et al.* Achieving ultrahigh energy storage performance over a broad temperature range in $(Bi_{0.5}Na_{0.5})TiO_3$-based eco-friendly relaxor ferroelectric ceramics via multiple engineering processes. *J. Alloys Compd.* **896**, 163139 (2022).
58. Qi, H. & Zuo, R. Linear-like lead-free relaxor antiferroelectric $(Bi_{0.5}Na_{0.5})TiO_3$–$NaNbO_3$ with giant energy-storage density/efficiency and super stability against temperature and frequency. *J. Mater. Chem. A* **7**, 3971–3978 (2019).
59. Yang, H., Cai, Z., Zhu, C., Feng, P. & Wang, X. Ultra-High Energy Storage Performance in BNT-based Ferroelectric Ceramics with Simultaneously Enhanced Polarization and Breakdown Strength. *ACS Sustain. Chem. Eng.* **10**, 9176–9183 (2022).
60. Zhang, C. *et al.* Superior energy-storage performance in $0.85Bi_{0.5}Na_{0.5}TiO_3$–$0.15NaNbO_3$ lead-free ferroelectric ceramics via composition and microstructure engineering. *J. Mater. Chem. A* **9**, 10088–10094 (2021).
61. Ji, H. *et al.* Ultrahigh energy density in short-range tilted NBT-based lead-free multilayer ceramic capacitors by nanodomain percolation. *Energy Storage Mater.* **38**, 113–120 (2021).
62. Wang, W. *et al.* Enhanced Energy Storage Properties in Lead-Free $(Na_{0.5}Bi_{0.5})_{0.7}Sr_{0.3}TiO_3$-Based Relaxor Ferroelectric Ceramics through a Cooperative Optimization Strategy. *ACS Appl. Mater. Interfaces* **15**, 6990–7001 (2023).
63. Ma, Q. *et al.* Excellent Energy-Storage Performance in Lead-Free Capacitors with Highly Dynamic Polarization Heterogeneous Nanoregions. *Small* **19**, 2303768 (2023).
64. Li, D. *et al.* Lead-Free Relaxor Ferroelectric Ceramics with Ultrahigh Energy Storage Densities via Polymorphic Polar Nanoregions Design. *Small* **19**, 2206958 (2023).
65. Zhang, L. *et al.* Enhanced antiferroelectric-like relaxor ferroelectric characteristic boosting energy storage performance of $(Bi_{0.5}Na_{0.5})TiO_3$-based ceramics *via* defect engineering. *J. Materiomics* **8**, 527–536 (2022).

66. Cao, W. *et al.* Boosting energy-storage performance in lead-free ceramics via polyphase engineering in the superparaelectric state. *Compos. Part B Eng.* **255**, 110630 (2023).
67. Guo, J. *et al.* Multi-symmetry high-entropy relaxor ferroelectric with giant capacitive energy storage. *Nano Energy* **112**, 108458 (2023).
68. Liu, H. *et al.* Chemical Design of Pb-Free Relaxors for Giant Capacitive Energy Storage. *J. Am. Chem. Soc.* **145**, 11764–11772 (2023).
69. Chen, L. *et al.* Large Energy Capacitive High-Entropy Lead-Free Ferroelectrics. *Nano-Micro Lett.* **15**, 65 (2023).
70. Cao, W. *et al.* Interfacial Polarization Restriction for Ultrahigh Energy-Storage Density in Lead-Free Ceramics. *Adv. Funct. Mater.* **33**, 2301027 (2023).
71. Liu, H. *et al.* Local Chemical Clustering Enabled Ultrahigh Capacitive Energy Storage in Pb-Free Relaxors. *J. Am. Chem. Soc.* **145**, 19396–19404 (2023).
72. Luo, H. *et al.* Outstanding Energy-Storage Density Together with Efficiency of above 90% via Local Structure Design. *J. Am. Chem. Soc.* **146**, 460–467 (2024).
73. Kang, R. *et al.* Superior energy storage capacity of a $Bi_{0.5}Na_{0.5}TiO_3$-based dielectric capacitor under moderate electric field by constructing multiscale polymorphic domains. *Nano Energy* **112**, 108477 (2023).
74. Chen, L. *et al.* Local Diverse Polarization Optimized Comprehensive Energy-Storage Performance in Lead-Free Superparaelectrics. *Adv. Mater.* **34**, 2205787 (2022).
75. Shi, P. *et al.* Tailoring ferroelectric polarization and relaxation of BNT-based lead-free relaxors for superior energy storage properties. *Chem. Eng. J.* **428**, 132612 (2022).
76. Wang, M. *et al.* Ultrahigh Energy Storage Density and Efficiency in $Bi_{0.5}Na_{0.5}TiO_3$-Based Ceramics via the Domain and Bandgap Engineering. *ACS Appl. Mater. Interfaces* **13**, 51218–51229 (2021).
77. Huang, Y.-N., Zhang, J., Wang, J., Wang, J. & Wang, Y. Ultrahigh energy storage density, high efficiency and superior thermal stability in $Bi_{0.5}Na_{0.5}TiO_3$ -based relaxor ferroelectric ceramics *via* constructing multiphase structures. *J. Mater. Chem. A* **11**, 7987–7994 (2023).
78. Tang, L. *et al.* Giant Energy Storage Density with Antiferroelectric-Like Properties in BNT-Based Ceramics via Phase Structure Engineering. *Small* **19**, 2302346 (2023).
79. Li, T. *et al.* Ultrahigh Energy-Storage Performances in Lead-free $Na_{0.5}Bi_{0.5}TiO_3$-Based Relaxor Antiferroelectric Ceramics through a Synergistic Design Strategy. *ACS Appl. Mater. Interfaces* **14**, 22263–22269 (2022).
80. Chu, B. *et al.* High-Energy Storage Properties over a Broad Temperature Range in La-Modified BNT-Based Lead-Free Ceramics. *ACS Appl. Mater. Interfaces* **14**, 19683–19696 (2022).
81. Cao, W. *et al.* Phase and Band Structure Engineering via Linear Additive in NBT-ST for Excellent Energy Storage Performance with Superior Thermal Stability. *ACS Appl. Mater. Interfaces* **14**, 54051–54062 (2022).
82. Zhu, X. *et al.* Ultrahigh energy storage density in $(Bi_{0.5}Na_{0.5})_{0.65}Sr_{0.35}TiO_3$-based lead-free relaxor ceramics with excellent temperature stability. *Nano Energy* **98**, 107276 (2022).
83. Zhao, W. *et al.* Broad-high operating temperature range and enhanced energy storage performances in lead-free ferroelectrics. *Nat. Commun.* **14**, 5725 (2023).
84. Shiga, M., Hagiwara, M. & Fujihara, S. $(Bi_{1/2}K_{1/2})TiO_3$–$SrTiO_3$ solid-solution ceramics for high-temperature capacitor applications. *Ceram. Int.* **46**, 10242–10249 (2020).
85. Li, F., Jiang, T., Zhai, J., Shen, B. & Zeng, H. Exploring novel bismuth-based materials for energy storage applications. *J. Mater. Chem. C* **6**, 7976–7981 (2018).

86. Zhao, P. *et al.* Improved dielectric breakdown strength and energy storage properties in Er_2O_3 modified $Sr_{0.35}Bi_{0.35}K_{0.25}TiO_3$. *Chem. Eng. J.* **403**, 126290 (2021).
87. Li, F., Si, R., Li, T., Wang, C. & Zhai, J. High energy storage performance and fast discharging speed in dense $0.7Bi_{0.5}K_{0.5}TiO_3$-$0.3SrTiO_3$ ceramics via a novel rolling technology. *Ceram. Int.* **46**, 6995–6998 (2020).
88. Yang, Q. *et al.* Excellent energy storage performance of $K_{0.5}Bi_{0.5}TiO_3$-based ferroelectric ceramics under low electric field. *Chem. Eng. J.* **414**, 128769 (2021).
89. Wei, Y. *et al.* A $Bi_{1/2}K_{1/2}TiO_3$-based ergodic relaxor ceramic for temperature-stable energy storage applications. *Mater. Des.* **207**, 109887 (2021).
90. Li, F. *et al.* Fine-grain induced outstanding energy storage performance in novel $Bi_{0.5}K_{0.5}TiO_3$–$Ba(Mg_{1/3}Nb_{2/3})O_3$ ceramics via a hot-pressing strategy. *J. Mater. Chem. C* **7**, 12127–12138 (2019).
91. Kang, R. *et al.* Enhanced energy storage performance of $Bi_{0.5}K_{0.5}TiO_3$-based ceramics via composition modulation. *J. Alloys Compd.* **935**, 167999 (2023).
92. Niu, Z. *et al.* $Bi_{0.5}K_{0.5}TiO_3$-based lead-free relaxor ferroelectric with high energy storage performances via the grain size and bandgap engineering. *Mater. Today Chem.* **24**, 100898 (2022).
93. Zheng, L. *et al.* Simultaneously achieving high energy storage performance and remarkable thermal stability in $Bi_{0.5}K_{0.5}TiO_3$-based ceramics. *Mater. Today Energy* **28**, 101078 (2022).
94. Wang, H. *et al.* Significantly Enhanced Energy Storage Performance in High Hardness BKT-Based Ceramic via Defect Engineering and Relaxor Tuning. *ACS Appl. Mater. Interfaces* **14**, 54021–54033 (2022).
95. Chen, L. *et al.* Outstanding Energy Storage Performance in High-Hardness $(Bi_{0.5}K_{0.5})TiO_3$-Based Lead-Free Relaxors via Multi-Scale Synergistic Design. *Adv. Funct. Mater.* **32**, 2110478 (2022).
96. Liu, H. *et al.* Chemical Framework to Design Linear-like Relaxors toward Capacitive Energy Storage. *J. Am. Chem. Soc.* **146**, 3498–3507 (2024).
97. Zhang, M. *et al.* Achieving excellent energy storage performance of $K_{1/2}Bi_{1/2}TiO_3$-based ceramics via multi-phase boundary and bandgap engineering. *Chem. Eng. J.* **473**, 145314 (2023).
98. Liu, W., Gao, J., Zhao, Y. & Li, S. Significant enhancement of energy storage properties of $BaTiO_3$-based ceramics by hybrid-doping. *J. Alloys Compd.* **843**, 155938 (2020).
99. Zhan, D. *et al.* Dielectric nonlinearity and electric breakdown behaviors of $Ba_{0.95}Ca_{0.05}Zr_{0.3}Ti_{0.7}O_3$ ceramics for energy storage utilizations. *J. Alloys Compd.* **682**, 594–600 (2016).
100. Shen, Z., Wang, X., Luo, B. & Li, L. $BaTiO_3$–$BiYbO_3$ perovskite materials for energy storage applications. *J. Mater. Chem. A* **3**, 18146–18153 (2015).
101. Yi, X. *et al.* Effects of Sintering Method and $BiAlO_3$ Dopant on Dielectric Relaxation and Energy Storage Properties of $BaTiO_3$–$BiYbO_3$ Ceramics. *Phys. Status Solidi A* **217**, 1900721 (2020).
102. Wang, T., Jin, L., Li, C., Hu, Q. & Wei, X. Relaxor Ferroelectric $BaTiO_3$–$Bi(Mg_{2/3}Nb_{1/3})O_3$ Ceramics for Energy Storage Application. *J. Am. Ceram. Soc.* **98**, 559–566 (2015).
103. Sun, Z., Li, L., Yu, S., Kang, X. & Chen, S. Energy storage properties and relaxor behavior of lead-free $Ba_{1-x}Sm_{2x/3}Zr_{0.15}Ti_{0.85}O_3$ ceramics. *Dalton Trans.* **46**, 14341–14347 (2017).
104. Jiang, X. *et al.* Enhanced energy storage and fast discharge properties of $BaTiO_3$ based ceramics modified by $Bi(Mg_{1/2}Zr_{1/2})O_3$. *J. Eur. Ceram. Soc.* **39**, 1103–1109 (2019).
105. Liu, X. *et al.* Enhanced energy storage properties of $BaTiO_3$-$Bi_{0.5}Na_{0.5}TiO_3$ lead-free ceramics modified by $SrY_{0.5}Nb_{0.5}O_3$. *J. Alloys Compd.* **778**, 97–104 (2019).
106. Zhu, C., Cai, Z., Li, L. & Wang, X. High energy density, high efficiency and excellent temperature stability of lead free Mn–doped $BaTiO_3$–$Bi(Mg_{1/2}Zr_{1/2})O_3$ ceramics sintered in a reducing atmosphere. *J.*

Alloys Compd. **816**, 152498 (2020).
107. Zhang, L., Pang, L.-X., Li, W.-B. & Zhou, D. Extreme high energy storage efficiency in perovskite structured (1-x)($Ba_{0.8}Sr_{0.2}$)TiO_3-xBi($Zn_{2/3}Nb_{1/3}$)O_3 (0.04 ≤ x ≤ 0.16) ceramics. *J. Eur. Ceram. Soc.* **40**, 3343–3347 (2020).
108. Li, W.-B., Zhou, D. & Pang, L.-X. Enhanced energy storage density by inducing defect dipoles in lead free relaxor ferroelectric $BaTiO_3$-based ceramics. *Appl. Phys. Lett.* **110**, 132902 (2017).
109. Li, Y. M. & Bian, J. J. Effects of reoxidation on the dielectric and energy storage properties of Ce-doped (Ba,Sr)TiO_3 ceramics prepared by hot-pressed sintering. *J. Eur. Ceram. Soc.* **40**, 5441–5449 (2020).
110. Hu, Q. *et al.* Dielectric and temperature stable energy storage properties of 0.88$BaTiO_3$–0.12Bi($Mg_{1/2}Ti_{1/2}$)O_3 bulk ceramics. *J. Alloys Compd.* **640**, 416–420 (2015).
111. Jain, A., Wang, Y. G., Wang, N. & Wang, F. L. Critical role of CuO doping on energy storage performance and electromechanical properties of $Ba_{0.8}Sr_{0.1}Ca_{0.1}Ti_{0.9}Zr_{0.1}O_3$ ceramics. *Ceram. Int.* **46**, 18800–18812 (2020).
112. Liu, G. *et al.* An investigation of the dielectric energy storage performance of Bi($Mg_{2/3}Nb_{1/3}$)O_3-modifed $BaTiO_3$ Pb-free bulk ceramics with improved temperature/frequency stability. *Ceram. Int.* **45**, 19189–19196 (2019).
113. Li, W.-B., Zhou, D. & Pang, L.-X. Structure and energy storage properties of Mn-doped (Ba,Sr)TiO_3–MgO composite ceramics. *J. Mater. Sci. Mater. Electron.* **28**, 8749–8754 (2017).
114. Dai, Z. *et al.* Enhanced energy storage properties and stability of Sr($Sc_{0.5}Nb_{0.5}$)O_3 modified 0.65$BaTiO_3$-0.35$Bi_{0.5}Na_{0.5}TiO_3$ ceramics. *Chem. Eng. J.* **397**, 125520 (2020).
115. Huang, Y., Zhao, C., Wu, B. & Wu, J. Multifunctional $BaTiO_3$-Based Relaxor Ferroelectrics toward Excellent Energy Storage Performance and Electrostrictive Strain Benefiting from Crossover Region. *ACS Appl. Mater. Interfaces* **12**, 23885–23895 (2020).
116. Zhou, M., Liang, R., Zhou, Z. & Dong, X. Combining high energy efficiency and fast charge-discharge capability in novel $BaTiO_3$-based relaxor ferroelectric ceramic for energy-storage. *Ceram. Int.* **45**, 3582–3590 (2019).
117. Zhou, M., Liang, R., Zhou, Z. & Dong, X. Novel $BaTiO_3$-based lead-free ceramic capacitors featuring high energy storage density, high power density, and excellent stability. *J. Mater. Chem. C* **6**, 8528–8537 (2018).
118. Si, F., Tang, B., Fang, Z., Li, H. & Zhang, S. A new type of $BaTiO_3$-based ceramics with Bi($Mg_{1/2}Sn_{1/2}$)O_3 modification showing improved energy storage properties and pulsed discharging performances. *J. Alloys Compd.* **819**, 153004 (2020).
119. Wang, Q., Gong, P.-M. & Wang, C.-M. High recoverable energy storage density and large energy efficiency simultaneously achieved in $BaTiO_3$–Bi($Zn_{1/2}Zr_{1/2}$)O_3 relaxor ferroelectrics. *Ceram. Int.* **46**, 22452–22459 (2020).
120. Chen, X. *et al.* Achieving ultrahigh energy storage density and energy efficiency simultaneously in barium titanate based ceramics. *Appl. Phys. A* **126**, 146 (2020).
121. Jain, A., Wang, Y. G. & Guo, H. Microstructural properties and ultrahigh energy storage density in $Ba_{0.9}Ca_{0.1}TiO_3$–$NaNb_{0.85}Ta_{0.15}O_3$ relaxor ceramics. *Ceram. Int.* **46**, 24333–24346 (2020).
122. Si, F., Tang, B., Fang, Z., Li, H. & Zhang, S. Enhanced energy storage and fast charge-discharge properties of (1-x)$BaTiO_3$-xBi($Ni_{1/2}Sn_{1/2}$)O_3 relaxor ferroelectric ceramics. *Ceram. Int.* **45**, 17580–17590 (2019).
123. Yuan, Q. *et al.* Simultaneously achieved temperature-insensitive high energy density and efficiency in domain engineered $BaTiO_3$-Bi($Mg_{0.5}Zr_{0.5}$)O_3 lead-free relaxor ferroelectrics. *Nano Energy* **52**, 203–

210 (2018).
124. Li, X., Chen, X., Sun, J., Zhou, M. & Zhou, H. Novel lead-free ceramic capacitors with high energy density and fast discharge performance. *Ceram. Int.* **46**, 3426–3432 (2020).
125. Huang, Q., Si, F. & Tang, B. The effect of rare-earth oxides on the energy storage performances in $BaTiO_3$ based ceramics. *Ceram. Int.* **48**, 17359–17368 (2022).
126. Wang, Y. *et al.* Structure, dielectric properties of novel $Ba(Zr,Ti)O_3$ based ceramics for energy storage application. *Ceram. Int.* **46**, 12080–12087 (2020).
127. Meng, D. *et al.* Realising high comprehensive energy storage performance of BaTiO3-based perovskite ceramics via $La(Zn_{1/2}Hf_{1/2})O_3$ modification. *Ceram. Int.* **48**, 16173–16182 (2022).
128. Chen, X. *et al.* Simultaneously achieving ultrahigh energy storage density and energy efficiency in barium titanate based ceramics. *Ceram. Int.* **46**, 2764–2771 (2020).
129. Dai, Z. *et al.* Effective Strategy to Achieve Excellent Energy Storage Properties in Lead-Free $BaTiO_3$-Based Bulk Ceramics. *ACS Appl. Mater. Interfaces* **12**, 30289–30296 (2020).
130. Liu, Z.-G., Li, M.-D., Tang, Z.-H. & Tang, X.-G. Enhanced energy storage density and efficiency in lead-free $Bi(Mg_{1/2}Hf_{1/2})O_3$-modified $BaTiO_3$ ceramics. *Chem. Eng. J.* **418**, 129379 (2021).
131. Liu, G. *et al.* Ultrahigh dielectric breakdown strength and excellent energy storage performance in lead-free barium titanate-based relaxor ferroelectric ceramics via a combined strategy of composition modification, viscous polymer processing, and liquid-phase sintering. *Chem. Eng. J.* **398**, 125625 (2020).
132. Liu, Z.-G. *et al.* Excellent energy storage density and efficiency in lead-free Sm-doped $BaTiO_3$–$Bi(Mg_{0.5}Ti_{0.5})O_3$ ceramics. *J. Mater. Chem. C* **8**, 13405–13414 (2020).
133. Hu, D. *et al.* Optimization the energy density and efficiency of $BaTiO_3$-based ceramics for capacitor applications. *Chem. Eng. J.* **409**, 127375 (2021).
134. Li, Y. *et al.* Energy storage performance of $BaTiO_3$-based relaxor ferroelectric ceramics prepared through a two-step process. *Chem. Eng. J.* **419**, 129673 (2021).
135. Dong, X., Li, X., Chen, X., Wu, J. & Zhou, H. Simultaneous enhancement of polarization and breakdown strength in lead-free $BaTiO_3$-based ceramics. *Chem. Eng. J.* **409**, 128231 (2021).
136. Hu, Q. *et al.* Achieve ultrahigh energy storage performance in $BaTiO_3$–$Bi(Mg_{1/2}Ti_{1/2})O_3$ relaxor ferroelectric ceramics via nano-scale polarization mismatch and reconstruction. *Nano Energy* **67**, 104264 (2020).
137. Huang, W. *et al.* Ultrahigh recoverable energy storage density and efficiency in barium strontium titanate-based lead-free relaxor ferroelectric ceramics. *Appl. Phys. Lett.* **113**, 203902 (2018).
138. Yang, H. *et al.* Novel $BaTiO_3$-Based, Ag/Pd-Compatible Lead-Free Relaxors with Superior Energy Storage Performance. *ACS Appl. Mater. Interfaces* **12**, 43942–43949 (2020).
139. Joseph, J., Cheng, Z. & Zhang, S. $NaNbO_3$ modified $BiScO_3$-$BaTiO_3$ dielectrics for high-temperature energy storage applications. *J. Materiomics* **8**, 731–738 (2022).
140. Yang, W. *et al.* Enhanced recoverable energy storage density and efficiency in $(1-x)Ba_{0.85}Ca_{0.15}Zr_{0.1}Ti_{0.9}O_3$-$xSrTiO_3$-$MnO_2$ lead-free ceramics. *J. Mater. Chem. C* **10**, 3876–3885 (2022).
141. Huang, W. *et al.* Superior energy storage performances achieved in $(Ba,Sr)TiO_3$-based bulk ceramics through composition design and Core-shell structure engineering. *Chem. Eng. J.* **444**, 135523 (2022).
142. Chen, X. *et al.* Ultrahigh energy density and efficiency of $BaTiO_3$-based ceramics via multiple design strategies. *Chem. Eng. J.* **467**, 143395 (2023).
143. Guo, J. *et al.* Achieving Excellent Energy Storage Properties in Fine-Grain High-Entropy Relaxor

Ferroelectric Ceramics. *Adv. Electron. Mater.* **8**, 2200503 (2022).
144. Long, C. *et al.* Simultaneously realizing ultrahigh energy storage density and efficiency in $BaTiO_3$-based dielectric ceramics by creating highly dynamic polar nanoregions and intrinsic conduction. *Acta Mater.* **256**, 119135 (2023).
145. Chen, L. *et al.* Excellent energy storage and mechanical performance in hetero-structure $BaTiO_3$-based relaxors. *Chem. Eng. J.* **452**, 139222 (2023).
146. Xiao, W. *et al.* Free energy regulation and domain engineering of $BaTiO_3$-$NaNbO_3$ ceramics for superior dielectric energy storage performance. *Chem. Eng. J.* **461**, 142070 (2023).
147. Li, D. *et al.* A high-temperature performing and near-zero energy loss lead-free ceramic capacitor. *Energy Environ. Sci.* **16**, 4511–4521 (2023).
148. Chen, L. *et al.* Near-Zero Energy Consumption Capacitors by Controlling Inhomogeneous Polarization Configuration. *Adv. Mater.* **36**, 2313285 (2024).
149. Liu, Z., Lu, J., Mao, Y., Ren, P. & Fan, H. Energy storage properties of $NaNbO_3$-$CaZrO_3$ ceramics with coexistence of ferroelectric and antiferroelectric phases. *J. Eur. Ceram. Soc.* **38**, 4939–4945 (2018).
150. Yang, Z. *et al.* A new family of sodium niobate-based dielectrics for electrical energy storage applications. *J. Eur. Ceram. Soc.* **39**, 2899–2907 (2019).
151. Xie, A., Qi, H. & Zuo, R. Achieving Remarkable Amplification of Energy-Storage Density in Two-Step Sintered $NaNbO_3$–$SrTiO_3$ Antiferroelectric Capacitors through Dual Adjustment of Local Heterogeneity and Grain Scale. *ACS Appl. Mater. Interfaces* **12**, 19467–19475 (2020).
152. Shi, R. *et al.* A novel lead-free $NaNbO_3$–$Bi(Zn_{0.5}Ti_{0.5})O_3$ ceramics system for energy storage application with excellent stability. *J. Alloys Compd.* **815**, 152356 (2020).
153. Fan, Y., Zhou, Z., Liang, R. & Dong, X. Designing novel lead-free $NaNbO_3$-based ceramic with superior comprehensive energy storage and discharge properties for dielectric capacitor applications via relaxor strategy. *J. Eur. Ceram. Soc.* **39**, 4770–4777 (2019).
154. Qu, N., Du, H. & Hao, X. A new strategy to realize high comprehensive energy storage properties in lead-free bulk ceramics. *J. Mater. Chem. C* **7**, 7993–8002 (2019).
155. Ye, J. *et al.* Excellent comprehensive energy storage properties of novel lead-free $NaNbO_3$-based ceramics for dielectric capacitor applications. *J. Mater. Chem. C* **7**, 5639–5645 (2019).
156. Zhou, M., Liang, R., Zhou, Z., Yan, S. & Dong, X. Novel Sodium Niobate-Based Lead-Free Ceramics as New Environment-Friendly Energy Storage Materials with High Energy Density, High Power Density, and Excellent Stability. *ACS Sustain. Chem. Eng.* **6**, 12755–12765 (2018).
157. Shi, J. *et al.* Superior thermal and frequency stability and decent fatigue endurance of high energy storage properties in $NaNbO_3$-based lead-free ceramics. *Ceram. Int.* **46**, 25731–25737 (2020).
158. Dong, X. *et al.* High energy storage and ultrafast discharge in $NaNbO_3$-based lead-free dielectric capacitors via a relaxor strategy. *Ceram. Int.* **47**, 3079–3088 (2021).
159. Zhou, M., Liang, R., Zhou, Z. & Dong, X. Achieving ultrahigh energy storage density and energy efficiency simultaneously in sodium niobate-based lead-free dielectric capacitors via microstructure modulation. *Inorg. Chem. Front.* **6**, 2148–2157 (2019).
160. Sun, C. *et al.* Simultaneously with large energy density and high efficiency achieved in $NaNbO_3$-based relaxor ferroelectric ceramics. *J. Eur. Ceram. Soc.* **41**, 1891–1903 (2021).
161. Yang, Z. *et al.* Realizing high comprehensive energy storage performance in lead-free bulk ceramics via designing an unmatched temperature range. *J. Mater. Chem. A* **7**, 27256–27266 (2019).
162. Zhou, M., Liang, R., Zhou, Z. & Dong, X. Superior energy storage properties and excellent stability of novel $NaNbO_3$-based lead-free ceramics with A-site vacancy obtained via a Bi_2O_3 substitution strategy.

J. Mater. Chem. A **6**, 17896–17904 (2018).
163. Wei, T. *et al.* Novel $NaNbO_3$-$Sr_{0.7}Bi_{0.2}TiO_3$ lead-free dielectric ceramics with excellent energy storage properties. *Ceram. Int.* **47**, 3713–3719 (2021).
164. Yang, L., Kong, X., Cheng, Z. & Zhang, S. Enhanced Energy Storage Performance of Sodium Niobate-Based Relaxor Dielectrics by a Ramp-to-Spike Sintering Profile. *ACS Appl. Mater. Interfaces* **12**, 32834–32841 (2020).
165. Chen, H. *et al.* Achieving ultrahigh energy storage density in $NaNbO_3$–$Bi(Ni_{0.5}Zr_{0.5})O_3$ solid solution by enhancing the breakdown electric field. *Ceram. Int.* **46**, 28407–28413 (2020).
166. Dong, X. *et al.* High energy storage density and power density achieved simultaneously in $NaNbO_3$-based lead-free ceramics via antiferroelectricity enhancement. *J. Materiomics* **7**, 629–639 (2021).
167. Shi, J. *et al.* Realizing ultrahigh recoverable energy density and superior charge–discharge performance in $NaNbO_3$-based lead-free ceramics via a local random field strategy. *J. Mater. Chem. C* **8**, 3784–3794 (2020).
168. Wang, X. *et al.* A Combined Optimization Strategy for Improvement of Comprehensive Energy Storage Performance in Sodium Niobate-Based Antiferroelectric Ceramics. *ACS Appl. Mater. Interfaces* **14**, 9330–9339 (2022).
169. Pang, F. *et al.* Ultrahigh Energy Storage Characteristics of Sodium Niobate-Based Ceramics by Introducing a Local Random Field. *ACS Sustain. Chem. Eng.* **8**, 14985–14995 (2020).
170. Tian, A., Zuo, R., Qi, H. & Shi, M. Large energy-storage density in transition-metal oxide modified $NaNbO_3$–$Bi(Mg_{0.5}Ti_{0.5})O_3$ lead-free ceramics through regulating the antiferroelectric phase structure. *J. Mater. Chem. A* **8**, 8352–8359 (2020).
171. Chen, H. *et al.* Enhanced thermal and frequency stability and decent fatigue endurance in lead-free NaNbO3-based ceramics with high energy storage density and efficiency. *J. Materiomics* **8**, 489–497 (2022).
172. Chen, J., Qi, H. & Zuo, R. Realizing Stable Relaxor Antiferroelectric and Superior Energy Storage Properties in $(Na_{1-x/2}La_{x/2})(Nb_{1-x}Ti_x)O_3$ Lead-Free Ceramics through A/B-Site Complex Substitution. *ACS Appl. Mater. Interfaces* **12**, 32871–32879 (2020).
173. Yang, L. *et al.* Excellent Energy Storage Properties Achieved in Sodium Niobate-Based Relaxor Ceramics through Doping Tantalum. *ACS Appl. Mater. Interfaces* **14**, 32218–32226 (2022).
174. Wu, S. *et al.* Superb Energy Storage Capability for $NaNbO_3$-Based Ceramics Featuring Labyrinthine Submicro-Domains with Clustered Lattice Distortions. *Small* **19**, 2303915 (2023).
175. Yang, W. *et al.* Superior energy storage properties in $NaNbO_3$ -based ceramics via synergistically optimizing domain and band structures. *J. Mater. Chem. A* **10**, 11613–11624 (2022).
176. Xu, Z. *et al.* Simultaneously achieving large energy density and high efficiency in $NaNbO_3$ -(Sr,Bi)TiO3-$Bi(Mg,Zr)O_3$ relaxor ferroelectric ceramics for dielectric capacitor applications. *J. Mater. Chem. A* **10**, 13907–13916 (2022).
177. Chen, H. *et al.* Excellent energy storage properties and stability of $NaNbO_3$–$Bi(Mg_{0.5}Ta_{0.5})O_3$ ceramics by introducing $(Bi_{0.5}Na_{0.5})_{0.7}Sr_{0.3}TiO_3$. *J. Mater. Chem. A* **9**, 4789–4799 (2021).
178. Xie, A. *et al.* $NaNbO_3$-$(Bi_{0.5}Li_{0.5})TiO_3$ Lead-Free Relaxor Ferroelectric Capacitors with Superior Energy-Storage Performances via Multiple Synergistic Design. *Adv. Energy Mater.* **11**, 2101378 (2021).
179. Chen, L. *et al.* Local Diverse Polarization Optimized Comprehensive Energy-Storage Performance in Lead-Free Superparaelectrics. *Adv. Mater.* **34**, 2205787 (2022).
180. Jiang, J. *et al.* Enhanced energy storage properties of lead-free $NaNbO_3$-based ceramics via A/B-site substitution. *Chem. Eng. J.* **422**, 130130 (2021).

181. Qi, H. *et al.* Ultrahigh Energy-Storage Density in $NaNbO_3$-Based Lead-Free Relaxor Antiferroelectric Ceramics with Nanoscale Domains. *Adv. Funct. Mater.* **29**, 1903877 (2019).
182. Pan, T. *et al.* Enhanced Energy Density and Efficiency in Lead-Free Sodium Niobate-Based Relaxor Antiferroelectric Ceramics for Electrostatic Energy Storage Application. *Adv. Electron. Mater.* **8**, 2200793 (2022).
183. Ma, J. *et al.* Achieving Ultrahigh Energy Storage Density in Lead-Free Sodium Niobate-Based Ceramics by Modulating the Antiferroelectric Phase. *Chem. Mater.* **34**, 7313–7322 (2022).
184. Jiang, J. *et al.* Novel lead-free $NaNbO_3$-based relaxor antiferroelectric ceramics with ultrahigh energy storage density and high efficiency. *J. Materiomics* **8**, 295–301 (2022).
185. Jiang, J. *et al.* Ultrahigh energy storage density in lead-free relaxor antiferroelectric ceramics via domain engineering. *Energy Storage Mater.* **43**, 383–390 (2021).
186. Bi, W. *et al.* Comprehensive energy-storage performance enhancement in relaxor anti-ferroelectrics via strengthening local polarization. *Chem. Eng. J.* **478**, 147383 (2023).
187. Gao, J. *et al.* Antiferroelectric-ferroelectric phase transition in lead-free $AgNbO_3$ ceramics for energy storage applications. *J. Am. Ceram. Soc.* **101**, 5443–5450 (2018).
188. Han, K. *et al.* Structure and energy storage performance of Ba-modified $AgNbO_3$ lead-free antiferroelectric ceramics. *Ceram. Int.* **45**, 5559–5565 (2019).
189. Zhao, L., Liu, Q., Zhang, S. & Li, J.-F. Lead-free $AgNbO_3$ anti-ferroelectric ceramics with an enhanced energy storage performance using MnO_2 modification. *J. Mater. Chem. C* **4**, 8380–8384 (2016).
190. Song, A. *et al.* Energy storage performance in $BiMnO_3$-modified $AgNbO_3$ anti-ferroelectric ceramics. *Mater. Lett.* **237**, 278–281 (2019).
191. Tian, Y. *et al.* Phase transitions in bismuth-modified silver niobate ceramics for high power energy storage. *J. Mater. Chem. A* **5**, 17525–17531 (2017).
192. Han, K. *et al.* Realizing high low-electric-field energy storage performance in $AgNbO_3$ ceramics by introducing relaxor behaviour. *J. Materiomics* **5**, 597–605 (2019).
193. Luo, N. *et al.* Lead-free $Ag_{1-3x}La_xNbO_3$ antiferroelectric ceramics with high-energy storage density and efficiency. *J. Am. Ceram. Soc.* **102**, 4640–4647 (2019).
194. Ren, P. *et al.* Grain size tailoring and enhanced energy storage properties of two-step sintered Nd^{3+}-doped $AgNbO_3$. *J. Eur. Ceram. Soc.* **40**, 4495–4502 (2020).
195. Zhao, L., Gao, J., Liu, Q., Zhang, S. & Li, J.-F. Silver Niobate Lead-Free Antiferroelectric Ceramics: Enhancing Energy Storage Density by B-Site Doping. *ACS Appl. Mater. Interfaces* **10**, 819–826 (2018).
196. Chao, W., Yang, T., Li, Y. & Liu, Z. Enhanced energy storage density in Ca and Ta co-doped $AgNbO_3$ antiferroelectric ceramics. *J. Am. Ceram. Soc.* **103**, 7283–7290 (2020).
197. Xu, Y. *et al.* High energy storage properties of lead-free Mn-doped $(1\text{-}x)AgNbO_3\text{-}xBi_{0.5}Na_{0.5}TiO_3$ antiferroelectric ceramics. *J. Eur. Ceram. Soc.* **40**, 56–62 (2020).
198. Mao, S. *et al.* Effect of Lu doping on the structure, electrical properties and energy storage performance of $AgNbO_3$ antiferroelectric ceramics. *J. Mater. Sci. Mater. Electron.* **31**, 7731–7741 (2020).
199. Feng, D. *et al.* Antiferroelectric stability and energy storage properties of Co-doped $AgNbO_3$ ceramics. *J. Solid State Chem.* **310**, 123081 (2022).
200. Xu, Y. *et al.* Enhanced energy density in Mn-doped $(1\text{-}x)AgNbO_3\text{-}xCaTiO_3$ lead-free antiferroelectric ceramics. *J. Alloys Compd.* **821**, 153260 (2020).
201. Zhao, L., Liu, Q., Gao, J., Zhang, S. & Li, J.-F. Lead-Free Antiferroelectric Silver Niobate Tantalate with High Energy Storage Performance. *Adv. Mater.* **29**, 1701824 (2017).
202. Gao, J. *et al.* Enhanced antiferroelectric phase stability in La-doped $AgNbO_3$: perspectives from

the microstructure to energy storage properties. *J. Mater. Chem. A* **7**, 2225–2232 (2019).
203. Gao, J. *et al.* Local Structure Heterogeneity in Sm-Doped $AgNbO_3$ for Improved Energy-Storage Performance. *ACS Appl. Mater. Interfaces* **12**, 6097–6104 (2020).
204. Li, S. *et al.* Significantly enhanced energy storage performance of rare-earth-modified silver niobate lead-free antiferroelectric ceramics via local chemical pressure tailoring. *J. Mater. Chem. C* **7**, 1551–1560 (2019).
205. Yan, Z. *et al.* Silver niobate based lead-free ceramics with high energy storage density. *J. Mater. Chem. A* **7**, 10702–10711 (2019).
206. Shi, P. *et al.* Significantly enhanced energy storage properties of Nd^{3+} doped $AgNbO_3$ lead-free antiferroelectric ceramics. *J. Alloys Compd.* **877**, 160162 (2021).
207. Luo, N. *et al.* Silver stoichiometry engineering: an alternative way to improve energy storage density of $AgNbO_3$-based antiferroelectric ceramics. *J. Mater. Res.* **36**, 1067–1075 (2021).
208. Han, K. *et al.* Ultrahigh energy-storage density in A-/B-site co-doped $AgNbO_3$ lead-free antiferroelectric ceramics: insight into the origin of antiferroelectricity. *J. Mater. Chem. A* **7**, 26293–26301 (2019).
209. Luo, N. *et al.* Aliovalent A-site engineered $AgNbO_3$ lead-free antiferroelectric ceramics toward superior energy storage density. *J. Mater. Chem. A* **7**, 14118–14128 (2019).
210. Luo, N. *et al.* Constructing phase boundary in $AgNbO_3$ antiferroelectrics: pathway simultaneously achieving high energy density and efficiency. *Nat. Commun.* **11**, 4824 (2020).
211. Lu, Z. *et al.* Mechanism of enhanced energy storage density in $AgNbO_3$-based lead-free antiferroelectrics. *Nano Energy* **79**, 105423 (2021).
212. Chao, W., Gao, J., Yang, T. & Li, Y. Excellent energy storage performance in La and Ta co-doped $AgNbO_3$ antiferroelectric ceramics. *J. Eur. Ceram. Soc.* **41**, 7670–7677 (2021).
213. Li, S. *et al.* Giant energy density and high efficiency achieved in silver niobate-based lead-free antiferroelectric ceramic capacitors *via* domain engineering. *Energy Storage Mater.* **34**, 417–426 (2021).
214. Zheng, D., Zuo, R., Zhang, D. & Li, Y. Novel $BiFeO_3$–$BaTiO_3$–$Ba(Mg_{1/3}Nb_{2/3})O_3$ Lead-Free Relaxor Ferroelectric Ceramics for Energy-Storage Capacitors. *J. Am. Ceram. Soc.* **98**, 2692–2695 (2015).
215. Zheng, D. & Zuo, R. Enhanced energy storage properties in $La(Mg_{1/2}Ti_{1/2})O_3$-modified $BiFeO_3$-$BaTiO_3$ lead-free relaxor ferroelectric ceramics within a wide temperature range. *J. Eur. Ceram. Soc.* **37**, 413–418 (2017).
216. Liu, N. *et al.* Novel bismuth ferrite-based lead-free ceramics with high energy and power density. *J. Am. Ceram. Soc.* **101**, 3259–3265 (2018).
217. Wang, D. *et al.* Bismuth ferrite-based lead-free ceramics and multilayers with high recoverable energy density. *J. Mater. Chem. A* **6**, 4133–4144 (2018).
218. Dabas, S., Kumar, M., Chaudhary, P. & Thakur, O. P. Enhanced magneto-electric coupling and energy storage analysis in Mn-modified lead free $BiFeO_3$-$BaTiO_3$ solid solutions. *J. Appl. Phys.* **126**, 134102 (2019).
219. Wang, D. *et al.* High Energy Storage Density and Large Strain in $Bi(Zn_{2/3}Nb_{1/3})O_3$-Doped $BiFeO_3$–$BaTiO_3$ Ceramics. *ACS Appl. Energy Mater.* **1**, 4403–4412 (2018).
220. Sun, H. *et al.* Large energy storage density in $BiFeO_3$-$BaTiO_3$-$AgNbO_3$ lead-free relaxor ceramics. *J. Eur. Ceram. Soc.* **40**, 2929–2935 (2020).
221. Chen, Z. *et al.* Simultaneously achieving high energy storage density and efficiency under low electric field in $BiFeO_3$-based lead-free relaxor ferroelectric ceramics. *J. Eur. Ceram. Soc.* **40**, 5450–

5457 (2020).
222. Liu, N., Liang, R., Zhou, Z. & Dong, X. Designing lead-free bismuth ferrite-based ceramics learning from relaxor ferroelectric behavior for simultaneous high energy density and efficiency under low electric field. *J. Mater. Chem. C* **6**, 10211–10217 (2018).
223. Chen, Z. *et al.* Achieving high-energy storage performance in 0.67$Bi_{1-x}Sm_xFeO_3$-0.33$BaTiO_3$ lead-free relaxor ferroelectric ceramics. *Ceram. Int.* **46**, 11549–11555 (2020).
224. Bai, X. *et al.* High recoverable energy storage density in nominal (0.67-x)$BiFeO_3$-0.33$BaTiO_3$-x$BaBi_2Nb_2O_9$ lead-free composite ceramics. *Ceram. Int.* **47**, 23116–23123 (2021).
225. Khesro, A. *et al.* Energy storage performance of Nd^{3+}-doped $BiFeO_3$–$BaTiO_3$-based lead-free ceramics. *Ceram. Int.* **48**, 29938–29943 (2022).
226. Yang, H., Qi, H. & Zuo, R. Enhanced breakdown strength and energy storage density in a new $BiFeO_3$-based ternary lead-free relaxor ferroelectric ceramic. *J. Eur. Ceram. Soc.* **39**, 2673–2679 (2019).
227. Wang, G. *et al.* Fatigue resistant lead-free multilayer ceramic capacitors with ultrahigh energy density. *J. Mater. Chem. A* **8**, 11414–11423 (2020).
228. Wang, M., Xie, A., Fu, J. & Zuo, R. Energy storage properties under moderate electric fields in $BiFeO_3$-based lead-free relaxor ferroelectric ceramics. *Chem. Eng. J.* **440**, 135789 (2022).
229. Guo, H. *et al.* Optimized energy storage performance in BF-BT-based lead-free ferroelectric ceramics with local compositional fluctuation. *J. Eur. Ceram. Soc.* **43**, 4774–4781 (2023).
230. Zhao, J. *et al.* Delayed Polarization Saturation Induced Superior Energy Storage Capability of BiFeO3-Based Ceramics Via Introduction of Non-Isovalent Ions. *Small* **19**, 2206840 (2023).
231. Qi, H., Xie, A., Tian, A. & Zuo, R. Superior Energy-Storage Capacitors with Simultaneously Giant Energy Density and Efficiency Using Nanodomain Engineered $BiFeO_3$-$BaTiO_3$-$NaNbO_3$ Lead-Free Bulk Ferroelectrics. *Adv. Energy Mater.* **10**, 1903338 (2020).
232. Lu, Z. *et al.* Superior energy density through tailored dopant strategies in multilayer ceramic capacitors. *Energy Environ. Sci.* **13**, 2938–2948 (2020).
233. Cui, T. *et al.* Simultaneous achievement of ultrahigh energy storage density and high efficiency in BiFeO3-based relaxor ferroelectric ceramics via a highly disordered multicomponent design. *J. Mater. Chem. A* **10**, 14316–14325 (2022).
234. Cui, T. Outstanding comprehensive energy storage performance in lead-free $BiFeO_3$-based relaxor ferroelectric ceramics by multiple optimization design. (2022).
235. Chai, Q., Yang, D., Zhao, X., Chao, X. & Yang, Z. Lead-free (K,Na)NbO_3-based ceramics with high optical transparency and large energy storage ability. *J. Am. Ceram. Soc.* **101**, 2321–2329 (2018).
236. Yang, Z. *et al.* Grain size engineered lead-free ceramics with both large energy storage density and ultrahigh mechanical properties. *Nano Energy* **58**, 768–777 (2019).
237. Qu, B., Du, H. & Yang, Z. Lead-free relaxor ferroelectric ceramics with high optical transparency and energy storage ability. *J. Mater. Chem. C* **4**, 1795–1803 (2016).
238. Zhang, M., Yang, H., Li, D. & Lin, Y. Excellent energy density and power density achieved in $K_{0.5}Na_{0.5}NbO_3$-based ceramics with high optical transparency. *J. Alloys Compd.* **829**, 154565 (2020).
239. Zhang, M., Yang, H., Li, D., Ma, L. & Lin, Y. Giant energy storage efficiency and high recoverable energy storage density achieved in $K_{0.5}Na_{0.5}NbO_3$-$Bi(Zn_{0.5}Zr_{0.5})O_3$ ceramics. *J. Mater. Chem. C* **8**, 8777–8785 (2020).
240. Xing, J. *et al.* Realizing High Comprehensive Energy Storage and Ultrahigh Hardness in Lead-Free Ceramics. *ACS Appl. Mater. Interfaces* **13**, 28472–28483 (2021).
241. Yang, Z. *et al.* Significantly enhanced recoverable energy storage density in potassium–sodium

niobate-based lead free ceramics. *J. Mater. Chem. A* **4**, 13778–13785 (2016).
242. Shao, T. *et al.* Potassium–sodium niobate based lead-free ceramics: novel electrical energy storage materials. *J. Mater. Chem. A* **5**, 554–563 (2017).
243. Zhang, Y. & Zuo, R. Excellent energy-storage performances in La2O3 doped (Na,K)NbO_3-based lead-free relaxor ferroelectrics. *J. Eur. Ceram. Soc.* **40**, 5466–5474 (2020).
244. Chen, B. *et al.* Ultrahigh storage density achieved with (1-x)KNN-xBZN ceramics. *J. Eur. Ceram. Soc.* **40**, 2936–2944 (2020).
245. Li, D. *et al.* Improved Energy Storage Properties Achieved in (K, Na)NbO_3-Based Relaxor Ferroelectric Ceramics via a Combinatorial Optimization Strategy. *Adv. Funct. Mater.* **32**, 2111776 (2022).
246. Zhang, M., Yang, H., Yu, Y. & Lin, Y. Energy storage performance of $K_{0.5}Na_{0.5}NbO_3$-based ceramics modified by $Bi(Zn_{2/3}(Nb_{0.85}Ta_{0.15})_{1/3})O_3$. *Chem. Eng. J.* **425**, 131465 (2021).
247. Zhang, M., Yang, H., Lin, Y., Yuan, Q. & Du, H. Significant increase in comprehensive energy storage performance of potassium sodium niobate-based ceramics via synergistic optimization strategy. *Energy Storage Mater.* **45**, 861–868 (2022).
248. Chen, L. *et al.* Giant energy-storage density with ultrahigh efficiency in lead-free relaxors via high-entropy design. *Nat. Commun.* **13**, 3089 (2022).
249. Xie, A. *et al.* Supercritical Relaxor Nanograined Ferroelectrics for Ultrahigh-Energy-Storage Capacitors. *Adv. Mater.* **34**, 2204356 (2022).
250. Yang, H. *et al.* A lead free relaxation and high energy storage efficiency ceramics for energy storage applications. *J. Alloys Compd.* **710**, 436–445 (2017).
251. Yang, H., Yan, F., Lin, Y. & Wang, T. Improvement of dielectric and energy storage properties in $SrTiO_3$-based lead-free ceramics. *J. Alloys Compd.* **728**, 780–787 (2017).
252. Cui, C. *et al.* Structure, dielectric and relaxor properties in lead-free ST-NBT ceramics for high energy storage applications. *J. Alloys Compd.* **711**, 319–326 (2017).
253. Cui, C., Pu, Y. & Shi, R. High-energy storage performance in lead-free (0.8-x)$SrTiO_3$-0.2$Na_{0.5}Bi_{0.5}TiO_{3\text{-}x}BaTiO_3$ relaxor ferroelectric ceramics. *J. Alloys Compd.* **740**, 1180–1187 (2018).
254. Yan, F., Yang, H., Lin, Y. & Wang, T. Dielectric and Ferroelectric Properties of $SrTiO_3$–$Bi_{0.5}Na_{0.5}TiO_3$–$BaAl_{0.5}Nb_{0.5}O_3$ Lead-Free Ceramics for High-Energy-Storage Applications. *Inorg. Chem.* **56**, 13510–13516 (2017).
255. Wang, W. *et al.* Enhanced energy storage density and high efficiency of lead-free $Ca_{1-x}Sr_xTi_{1-y}Zr_yO_3$ linear dielectric ceramics. *J. Eur. Ceram. Soc.* **39**, 5236–5242 (2019).
256. Wang, W. *et al.* Combining high energy efficiency and fast charge-discharge capability in calcium strontium titanate-based linear dielectric ceramic for energy-storage. *Ceram. Int.* **46**, 11484–11491 (2020).
257. Zhao, P. *et al.* Novel Ca doped Sr0.7Bi0.2TiO3 lead-free relaxor ferroelectrics with high energy density and efficiency. *J. Eur. Ceram. Soc.* **40**, 1938–1946 (2020).
258. Zhang, L. *et al.* Enhanced energy storage performance in Sn doped $Sr_{0.6}(Na_{0.5}Bi_{0.5})_{0.4}TiO_3$ lead-free relaxor ferroelectric ceramics. *J. Eur. Ceram. Soc.* **39**, 3057–3063 (2019).
259. Pan, W. *et al.* High breakdown strength and energy storage performance in (Nb, Zn) modified $SrTiO_3$ ceramics via synergy manipulation. *J. Mater. Chem. C* **8**, 2019–2027 (2020).
260. Yang, H., Yan, F., Lin, Y. & Wang, T. Enhanced recoverable energy storage density and high efficiency of $SrTiO_3$-based lead-free ceramics. *Appl. Phys. Lett.* **111**, 253903 (2017).
261. Yang, H., Yan, F., Lin, Y. & Wang, T. Novel Strontium Titanate-Based Lead-Free Ceramics for

High-Energy Storage Applications. *ACS Sustain. Chem. Eng.* **5**, 10215–10222 (2017).
262. Cui, C., Pu, Y. Improvement of energy storage density with trace amounts of ZrO_2 additives fabricated by wet-chemical method. *J. Alloys Compd.* **747**, 495–504 (2018).
263. Kong, X., Yang, L., Cheng, Z. & Zhang, S. Bi-modified $SrTiO_3$-based ceramics for high-temperature energy storage applications. *J. Am. Ceram. Soc.* **103**, 1722–1731 (2020).
264. Pu, Y. *et al.* Enhancing the energy storage properties of $Ca_{0.5}Sr_{0.5}TiO_3$-based lead-free linear dielectric ceramics with excellent stability through regulating grain boundary defects. *J. Mater. Chem. C* **7**, 14384–14393 (2019).
265. Guo, X. *et al.* Ultrahigh energy storage performance and fast charge-discharge capability in Dy-modified $SrTiO_3$ linear ceramics with high optical transmissivity by defect and interface engineering. *Ceram. Int.* **46**, 21719–21727 (2020).
266. Kong, X., Yang, L., Cheng, Z. & Zhang, S. Ultrahigh Energy Storage Properties in $(Sr_{0.7}Bi_{0.2})TiO_3$-$Bi(Mg_{0.5}Zr_{0.5})O_3$ Lead-Free Ceramics and Potential for High-Temperature Capacitors. *Materials* **13**, 180 (2020).
267. Zuo, C., Yang, S., Cao, Z., Yu, H. & Wei, X. Excellent energy storage and hardness performance of Sr0.7Bi0.2TiO3 ceramics fabricated by solution combustion-synthesized nanopowders. *Chem. Eng. J.* **442**, 136330 (2022).
268. Liu, L. Multi-scale collaborative optimization of $SrTiO_3$-based energy storage ceramics with high performance and excellent stability. *Nano Energy* (2023).
269. Cui, T. *et al.* Energy storage performance of $BiFeO_3$–$SrTiO_3$–$BaTiO_3$ relaxor ferroelectric ceramics. *J. Am. Ceram. Soc.* **105**, 6252–6261 (2022).
270. Yan, F. *et al.* Composition and Structure Optimized $BiFeO_3$-$SrTiO_3$ Lead-Free Ceramics with Ultrahigh Energy Storage Performance. *Small* **18**, 2106515 (2022).
271. Peng, H. *et al.* High-entropy relaxor ferroelectric ceramics for ultrahigh energy storage. *Nat. Commun.* **15**, 5232 (2024).
272. Gao, Y. *et al.* Optimizing high-temperature energy storage in tungsten bronze-structured ceramics via high-entropy strategy and bandgap engineering. *Nat. Commun.* **15**, 5869 (2024).
273. Gao, Y. *et al.* Ultrahigh Energy Storage in Tungsten Bronze Dielectric Ceramics Through a Weakly Coupled Relaxor Design. *Adv. Mater.* **36**, 2310559 (2024).
274. Gao, L. *et al.* Inhibiting oxygen vacancies and twisting NbO6 octahedron in erbium modified KNN-based multifunctional ceramics. *J. Materiomics.* **10**, 179-189 (2024).
275. Pu, Y. *et al.* Dielectric properties and electrocaloric effect of high-entropy $(Na_{0.2}Bi_{0.2}Ba_{0.2}Sr_{0.2}Ca_{0.2})TiO_3$ ceramic. APPL. PHYS. LETT. **115**, 223901 (2019).
276. Hussain, A. *et al.* High-entropic relaxor ferroelectric perovskites ceramics with A-site modification for energy storage applications. *Ceram. Int.* **49**, 39419-39427 (2023).
277. Yang, W., Zheng, G. Tunning the dielectric and energy storage properties of high entropy ceramics $(Bi_{0.2}Na_{0.2}K_{0.2}La_{0.2}Sr_{0.2})(Ti_{1-x}Sc_x)O_3$ by Sc-doping at B-site in perovskite structure. *J. Electroceram.* **49**, 53-62 (2022).
278. Wang, T. *et al.* Simultaneous excellent energy storage density and efficiency under applied low electric field for high entropy relaxor ferroelectric ceramics. Mater. Res. Bull. **157**, 112024 (2023).
279. Sun, W. *et al.* Enhanced electrical properties of $(Bi_{0.2}Na_{0.2}Ba_{0.2}Ca_{0.2}Sr_{0.2})TiO_3$ high-entropy ceramics prepared by hydrothermal method. *Ceram. Int.* **48**, 19492-19500 (2022).
280. Zhu, X. *et al.* Dielectric properties and excellent energy storage density under low electric fields for high entropy relaxor ferroelectric $(Li_{0.2}Ca_{0.2}Sr_{0.2}Ba_{0.2}La_{0.2})TiO_3$ ceramic. *J. Alloys Compd.* **984**, 173987

(2024).
281. Fang, J. *et al.* Energy storage properties of Mn-modified ($Na_{0.2}Bi_{0.2}Ca_{0.2}Sr_{0.2}Ba_{0.2}$)$TiO_3$ high-entropy relaxor-ferroelectric ceramics. *Results Phys.* ***3*8**, 105617 (2022).
282. Liu, J. *et al.* Structure, dielectric, and relaxor properties of $BaTiO_3$-modified high-entropy ($Bi_{0.2}Na_{0.2}K_{0.2}Ba_{0.2}Ca_{0.2}$)$TiO_3$ ceramics for energy storage applications. *J. Alloys Compd.* **947**, 169626 (2023).
283. Zhang, X. *et al.* Effect of La substitution on energy storage properties of ($Bi_{0.2}Na_{0.2}Ca_{0.2}Ba_{0.2}Sr_{0.2}$)$TiO_3$ lead-free high-entropy ceramics. *J. Am. Ceram. Soc.* **106**, 6641-6653 (2023).
284. Ye, W. *et al.* Enhanced energy-storage properties in Zr4+-modified ($Bi_{0.4}Ba_{0.2}K_{0.2}Na_{0.2}$)$TiO_3$ high-entropy ceramics. *J. Am. Ceram. Soc.* **106**, 6858-6867 (2023).
285. Lu, Y. *et al.* SPS-Prepared High-Entropy ($Bi_{0.2}Na_{0.2}Sr_{0.2}Ba_{0.2}Ca_{0.2}$)$TiO_3$ Lead-Free Relaxor-Ferroelectric Ceramics with High Energy Storage Density. CRYSTALS **13**, 445 (2023).
286. Liu, T. *et al.* Screening sintering aids for 0.88($Bi_{0.4}Na_{0.2}K_{0.2}Ba_{0.2}$)$TiO_3$-0.12Sr($Mg_{1/3}Nb_{2/3}$)$O_3$ high-entropy dielectric ceramics. *J. Am. Ceram. Soc.* **106**, 836-840 (2023).
287. Yang, T. *et al.* Enhanced energy storage performance in Bi($Mg_{1/3}Zn_{1/3}Ta_{1/3}$)O_3-doped ($K_{1/2}Na_{1/2}$)NbO_3 high-entropy ceramics. *Ceram. Int.* **49**, 36173-36180 (2023).
288. Ning, Y. *et al.* Achieving high energy storage performance below 200 kV/cm in $BaTiO_3$-based medium-entropy ceramics. *Ceram. Int.* **49**, 20326-20333 (2023).
289. Yan, B. *et al.* Design and preparation of lead-free ($Bi_{0.4}Na_{0.2}K_{0.2}Ba_{0.2}$)$TiO_3$-Sr($Mg_{1/3}Nb_{2/3}$)$O_3$ high-entropy relaxor ceramics for dielectric energy storage. *Chem. Eng. J.* **453**, 139921 (2023).
290. Yan, B. *et al.* Design and energy storage performance of ($Bi_{0.4}K_{0.2}Na_{0.2}Ba_{0.2}$)$TiO_3$-Sr ($Mg_{1/3}Ta_{2/3}$)$O_3$ high-entropy Relaxor ceramics. Mater. Res. Bull. **167**, 112392 (2023).
291. Ning, Y. *et al.* Enhanced capacitive energy storage and dielectric temperature stability of A-site disordered high-entropy perovskite oxides. *J. Mater. Sci. Technol.* **145**, 66-73 (2023).
292. Zhou, S. *et al.* Dielectric temperature stability and energy storage performance of NBT-based ceramics by introducing high-entropy oxide. *J. Am. Ceram. Soc.* **105**, 4796-4804 (2022).
293. Si, H. *et al.* Improving the energy storage performance of 0.88($Bi_{0.4}Ba_{0.2}Na_{0.2}K_{0.2}$) TiO_3-0.12Sr($Mg_{1/3}Nb_{2/3}$)O_3 high-entropy relaxor ceramics by AlN doping. *Ceram. Int.* **49**, 25179-25184 (2023).
294. Ning, Y. *et al.* Achieving high energy storage properties in perovskite oxide via high-entropy design. *Ceram. Int.* **49**, 12214-12223 (2023).
295. Zuo, C. *et al.* Combustion synthesis of high-performance high-entropy dielectric ceramics for energy storage applications. *Ceram. Int.* **49**, 25486-25494 (2023).
296. Li, Z. *et al.* Enhanced energy storage performance of $BaTi_{0.97}Ca_{0.03}O_{2.97}$-based ceramics by doping high-entropy perovskite oxide. *J. Alloys Compd.* **922**, 166179 (2022).
297. Tang, X. *et al.* High energy storage density under low electric fields in $BiFeO_3$-based ceramics with max configurational entropy. *J. Alloys Compd.* **43**, 6875-6882 (2023).
298. Bai, M. *et al.* High-performance energy storage in $BaTiO_3$-based oxide ceramics achieved by high-entropy engineering. *J. Alloys Compd.* **970**, 172671 (2024).
299. Sun, X. *et al.* The enhancement of energy storage performance in high-entropy ceramic. *Ceram. Int.* **49**, 17091-17098 (2023).
300. Ning, Y. *et al.* Remarkable energy-storage density together with efficiency of above 92% in high-entropy ferroelectric ceramics. Mat. Today Phys. **43**, 101418 (2024).
301. Chen, Z. *et al.* Entropy regulation enhanced superior energy storage density and high temperature

stability in lead-free relaxors. *J. Eur. Ceram. Soc.* **44**, 4680-4688 (2024).
302. Niu, Y. *et al.* High energy storage properties achieved in 0.94($Bi_{0.5}Na_{0.5}$)TiO_3-0.06$BaTiO_3$ based relaxor ferroelectric ceramics via high-entropy design. *Ceram. Int.* 5**0**, 22662-22670 (2024).
303. Chen, Z. *et al.* Prominent energy storage density and efficiency of $Na_{0.5}Bi_{0.5}TiO_3$-based ceramics via multiscale amelioration strategy. *J. Am. Ceram. Soc.* **107**, 2360-2370 (2023).
304. Qi, J. *et al.* High-entropy assisted $BaTiO_3$-based ceramic capacitors for energy storage. *Cell Rep. Phys. Sci.* **3**, 101110 (2022).
305. Ning, Y. *et al.* Novel high-entropy relaxors with ultrahigh energy-storage efficiency and density. *Chem. Eng. J.* **476**, 146673 (2023).
306. Wang, Z. *et al.* Ultrahigh energy-storage capacity achieved in ($Bi_{0.5}Na_{0.5}$)TiO_3-based high-entropy dielectric capacitors with linear-like polarization response. *Chem. Eng. J.* **474**, 145506 (2023).
307. Chen, Z. *et al.* Remarkable energy storage capability and dielectric temperature stability in ($Na_{0.5}Bi_{0.5}$)TiO_3-based medium entropy superparaelectrics. *Chem. Eng. J.* **481**, 148796 (2024).
308. Zhao, H. *et al.* Lead-free medium-entropy ($Na_{0.47(1-x)}Bi_{0.47(1-x)}Ba_{0.06(1-x)}Sr_{0.7x}Nd_{0.2x}$)$TiO_3$ relaxor ceramics with robust energy-storage performance. *Chem. Eng. J.* **471**, 144702 (2023).
309. Wang, H. *et al.* ($Bi_{1/6}Na_{1/6}Ba_{1/6}Sr_{1/6}Ca_{1/6}Pb_{1/6}$)$TiO_3$-based high-entropy dielectric ceramics with ultrahigh recoverable energy density and high energy storage efficiency. *J. Mater. Chem. A* **11**, 4937–4945, (2023).
310. Zhao, H. *et al.* Suppressing interfacial polarization via entropy increase strategy for superior energy-storage performance of $Na_{0.5}Bi_{0.5}TiO_3$-based ceramics. *J. Materiomics* **10**, 947-955 (2024).
311. Duan, J. *et al.* High-Entropy Tungsten Bronze Ceramics for Large Capacitive Energy Storage with Near-Zero Losses. *Adv. Funct. Mater.* **34**, 2409446 (2024).
312. Chen, L. *et al.* Large Energy Capacitive High-Entropy Lead-Free Ferroelectrics. *Nano-Micro Letters* **15**, 65 (2023).
